# A Series of Cube-Shaped Polyoxoniobates Encapsulating Octahedral Cu_12_X_**m**_O_**n**_ Clusters With Hydrolytic Decomposition for Chemical Warfare Agents

**DOI:** 10.3389/fchem.2020.586009

**Published:** 2020-12-18

**Authors:** Yan-Lan Wu, Rong-Tao Zhang, Yan-Qiong Sun, Xin-Xiong Li, Shou-Tian Zheng

**Affiliations:** State Key Laboratory of Photocatalysis on Energy and Environment, College of Chemistry, Fuzhou University, Fuzhou, China

**Keywords:** polyoxoniobates, cube-shaped, three shell, base-catalysis, chemical warfare agent simulant

## Abstract

This study reported a series of cube-shaped polyoxoniobates, {MCu_12_O_8_)(Cu_12_X_m_O_n_)(Nb_7_(OH)O_21_)_8_} [M = Nb(1, 2), Ln^3+^(3), X = I(1, *m* = 3, *n* = 3; 2, *m* = 5, *n* = 1), Br(3, *m* = 5, *n* = 1)]. As the first octahedral Cu_12_X_m_O_n_ cluster incorporated polyoxoniobate, the cube-shaped three-shell structure of {MCu_12_O_8_)(Cu_12_X_m_O_n_)(Nb_7_(OH)O_21_)_8_} polyanion contains a {MCu_12_O_8_} body-centered cuboctahedron, a {Cu_12_X_m_O_n_} octahedron and a {Cu_12_(Nb_7_(OH)O_21_)_8_} cube. Compounds 1, 2, 3 show effective catalytic activities for the hydrolytic decomposition of chemical warefare agent simulants.

## Introduction

Polyoxoniobates (PONbs) have obtained increasing attention in the past few years due to their well-defined structures and potential applications in photocatalysis (Zhang et al., 2011; Huang et al., 2012b; Qiao et al., 2018), catalysis (Ivanchikova et al., 2014; Zhu et al., 2020), virology (Judd et al., 2001; Wang et al., 2019), and nuclear waste treatment (Bonhomme et al., 2005). Although there has been a great deal of research exploring the PONbs in recent decades, developments lag far behind those of polyoxotungstates, polyoxomolybdates, and polyoxovanadates due to a lack of soluble niobate oxoanion precursors, low activity, and the narrow working pH region of the niobate species. A series of isopolyoxoniobates, including {Nb_10_} (Shen et al., 2008), {Nb_16_} (Liang et al., 2019), {Nb_20_} (Maekaw et al., 2006; Liang et al., 2019), {Nb_24_} (Bontchev and Nyman, 2006), {Nb_27_} (Tsunashima et al., 2010), {Nb_31_} (Tsunashima et al., 2010), {Nb_32_} (Huang et al., 2012a), {Nb_47_}(Wu et al., 2018), {Nb_52_} (Jin et al., 2017), {Nb_81_} (Jin et al., 2017), {Nb_114_} (Jin et al., 2017), and the highest nuclear PONb {Nb_288_} (Wu et al., 2018) were recently successfully synthesized. Since the first example of a heteropolyniobate Keggin-type structure, {[Ti_2_O_2_][SiNb_12_O_40_]} was identified by Nyman et al. (2002), and transition-metal-incorporated PONbs, such as {Cu_25.5_Nb_56_} (Niu et al., 2007), {Co_14_Nb_56_} (Niu et al., 2014), {Ti_12_Nb_6_} (Ohlin et al., 2008), {Fe_3_Nb_25_} (Liang et al., 2017), {Cr_2.5_Nb_27.5_} (Guo et al., 2019), and lanthanide-containing PONbs {Ln_12_W_12_Nb_72_} (Ln = Y, La, Sm, Eu, Yb) (Jin et al., 2016) were also reported in the past few years. However, most reports use transition metals or lanthanide cations as links to bridge PONb clusters, and very few polynuclear transition metals or lanthanide clusters are incorporated in PONbs. More recently, vanadium-cluster-substituted polyoxoniobates {V_x_Nb_y_O_z_} were characterized in studies by Huang et al. (2012a) and Hu et al. (2016, 2017). These indicate that combining a polynuclear transition metal cluster with typical PONbs increases the diversities of PONbs chemistry, which offers new strategies for designing and synthesizing functional materials.

Materials based on a cupreous-halide cluster with abundant structures have been applied to a colorimetric sensor (Yu et al., 2014), thermochromism (Kim et al., 2008), near-IR devices (Shan et al., 2013), and photoluminescence materials (Lee et al., 2008). Although many cupreous-halide clusters are incorporated into metal-organic frameworks (MOFs) (Kim et al., 2008; Lee et al., 2008; Shan et al., 2013; Yu et al., 2014) cupreous-halide clusters are not introduced to polyoxoniobates until now. The main limitation in the development of heteropolyoxoniobates is the sensitivity of their synthetic conditions. It is thus challenging to synthesize cupreous-halid-cluster incorporated PONbs. Based on this consideration, this study aims to combine the cupreous-halide cluster with polyoxoniobates to construct novel heteropolyoxoniobates.

Chemical warfare agents (CWAs), as a class of chemical weapons (Dong et al., 2017), have been the subject of recent studies, which have explored materials that are effective in facilitating hydrolytic decomposition for CWAs. A series of Zr-based MOFs were originally applied to degrade these toxic CWAs, including dimethyl 4-nitrophenyl phosphate (DMNP) (Zhao et al., 2016), 2-chloroethylethylsulfide (CEES) (López-Maya et al., 2015), diisopropylfluorophosphate (DIFP) (López-Maya et al., 2015), but there are limited papers on the hydrolysis of diethyl cyanophonate (DECP) and dimethyl methylphosphonate (DMMP) (López-Maya et al., 2015). Since Guo et al. (2016) reported the decontamination of DMMP and DECP by using PONbs as a catalyst, PONbs have been regarded as effective catalysts to hydrolyze chemicals and warfare agent simulants.

However, new materials and methods that rapidly and fully degrade all the main CWAs under mild conditions require further investigation.

Based on our ongoing research on PONbs, this study reports on three novel cupreous-halide incorporated PONbs, Na_12_{H_24_NbO_8_Cu_24_I_3_O_3_[Nb_7_(OH)O_21_]_8_}·34H_2_O (**1**), Na_12_(H_2_O)_22_{H_22_NbO_8_Cu_24_I_5_O[Nb_7_(OH)O_21_]_8_}·34H_2_O (**2**), Na_2_{H_34_GdO_8_Cu_24_Br_5_O[Nb_7_(OH)O_21_]_8_}·12H_2_O (**3**), which are the first examples of cupreous-halide-incorporated polyoxoniobate reported to date. The {MCu_12_O_8_)(Cu_12_X_m_O_n_)(Nb_7_(OH)-O_21_)_8_} polyanion possesses a three-shell cube-shaped structure that encapsulated the {Cu_12_X_m_O_n_} octahedron and {MCu_12_O_8_} body-centered cuboctahedron. Interestingly, this approach can effectively vary body-centered metal atoms. Base-catalysis studies reveal that **1** is an effective catalyst for the decomposition of DMMP and DECP.

## Results and Discussion

Single crystal X-ray diffraction reveals the brown compound **1** crystallizes in triclinic *P*$\bar{1}$ space group. Compound **1** possesses a 3D extended inorganic polyoxometalates framework constructed from cupreous-halide incorporated {(NbO_8_Cu_24_I_3_O_3_)(Nb_7_(OH)O_21_)_8_}^36−^ polyanions linked by Na^+^ bridges. The structure of {(NbO_8_Cu_24_I_3_O_3_)(Nb_7_(OH)-O_21_)_8_}^36−^ polyanion is a multimeric assembly of a body-centered cuboctahedral {NbCu_12_O_8_} cage-cluster unit with a disorder distribution of Nb(V) cation in the center, an octahedral {Cu_12_I_3_O_3_} and eight {Nb_7_(OH)O_21_}^8−^ cluster subunits.

**Figure 1:**
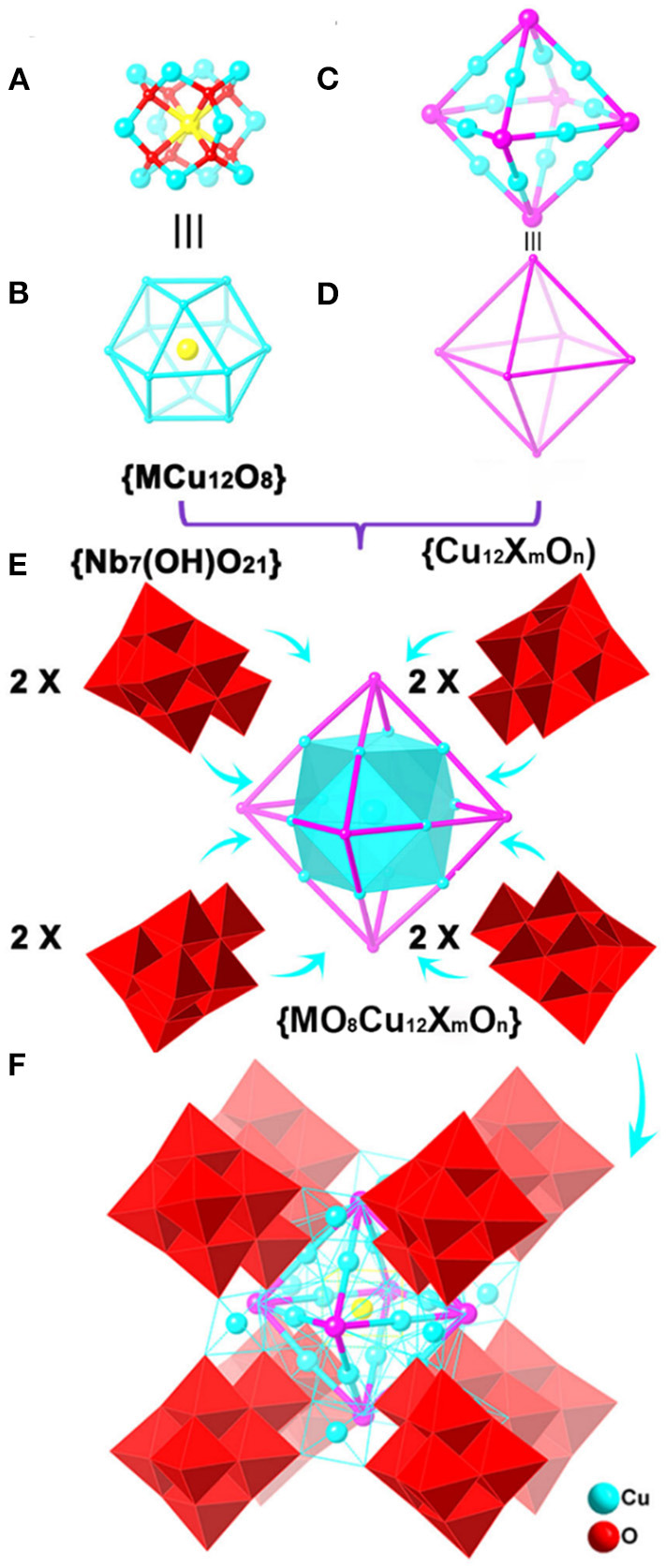
The structure assembly of {(MCu_12_O_8_)(Cu_12_X_m_O_n_)(Nb_7_(OH)O_21_)_8_} polyanion in compounds **1**, **2**, and **3**. **(A)** {MCu_12_O_8_} cluster unit [M = Nb (**1**), Nb (**2**), Ln (**3**)], **(B)** the topology of {MCu_12_O_8_} cluster unit [M = Nb (**1**), Nb (**2**), Ln (**3**)], **(C)** the motif of {Cu_12_X_m_O_n_} [X = I (**1**, *m* = 3, *n* = 3; **2**, *m* = 5, *n* = 1), Br (**3**, *m* = 5, *n* = 1)], **(D)** the topology of motif {Cu_12_X_m_O_n_}, **(E)** the assembly of {MO_8_Cu_12_X_m_O_n_} cluster and {Nb_7_(OH)O_21_} clusters, **(F)** the structure of the polyanion, Color code: M, yellow; X/O, purple; [Nb_7_(OH)O_21_]_8_: red polyhedral.

The {(NbO_8_Cu_24_I_3_O_3_)(Nb_7_(OH)O_21_)_8_}^36−^ polyanion in **1** possesses a centrosymmetric structure, which can be described as a three-shell structure. The innermost shell is a body-centered cuboctahedral {NbCu_12_O_8_} cluster unit (Figure 1A). The Nb atom located in the center of the cuboctahedron adopts special eight-coordinated cube geometry. In the {NbO_8_} core, the central Nb atom is disordered at seven positions, and the site occupancies of Nb_3A_, Nb_3A_'(1-x, 1-y, −4-z), Nb_4A_, Nb_4A_'(1-x, 1-y, −4-z), Nb_5A_, Nb_5A_'(1-x, 1-y, −4-z), Nb_37_ atoms are 0.120, 0.120, 0.126, 0.126, 0.128, 0.128, and 0.252, respectively. There is, therefore, one Nb atom in the central position. The Nb-O bond lengths are located in the range of 1.965 (6)−2.421 (4) Å (Supplementary Figure 1). This phenomenon is probably attributed to the steric effect because the Nb atom is located in the central of the regular cuboctahedron. Bond-valences sum calculations (BVS) show that the valance states of the Cu atoms are +2, which is verified by XPS analysis (Supplementary Figure 2). As shown in Figures 1A,C, 12 Cu atoms are linked by eight μ_4_-O atoms to form a 13-nuclearity body-centered cuboctahedron {NbCu_12_O_8_} cage-cluster unit containing six Cu_4_ square and eight triangular Cu_3_ windows. It is noteworthy that such a body-centered cuboctahedron {NbCu_12_O_8_} cage-cluster is rarely found in polyoxoniobates.

The second shell is an octahedral {Cu_12_I_3_O_3_} cage-cluster, with six disordered iodine/oxygen bridges (Figures 1B,D). Each iodine/oxygen site is shared by iodine and oxygen atoms simultaneously. The site occupancies of I_1_, I_1_' (1-x, 1-y, −4-z), I_2_, I_2_' (1-x, 1-y, −4-z), I_3_, I_3_' (1-x, 1-y, −4-z) anions are 0.55, 0.55, 0.5, 0.5, 0.45, and 0.45, respectively, while the shared site occupancies of O_1M_, O_1M_' (1-x, 1-y, −4-z), O_2M_, O_2M_' (1-x, 1-y, −4-z), O_3M_, O_3M_' (1-x, 1-y, −4-z) anions are 0.45, 0.45, 0.5, 0.5, 0.55, and 0.55, respectively. Every Cu^2+^ cation is six-coordinated by four bridged O atoms with Cu-O bond distances in the range of 1.905 (1)−2.003 (1) Å, and 2 μ_4_-I/O atoms with Cu-I/O bond lengths ranging from 3.091 (1) to 3.331 (1) Å, forming an elongated {CuO_4_I_2_} octahedron (Supplementary Figure 1). The Cu-I bond lengths are slightly longer than those Cu-I (2.920 (5)−3.140 (5) Å) of cupreous-halid MOFs (Zhao et al., 2017), but shorter than dissociative iodine chain [3.570 (1) Å] in many organic frameworks (Pantenburg and Müller, 2004). The six I/O atoms are arranged in the vertex while 12 Cu^2+^ cations are located at the midpoint of the 12 edges of the octahedron. Every I/O atom is connected with four Cu^2+^ cations, resulting in the formation of the {Cu_12_I_3_O_3_} octahedron as the second shell (Figures 1A–D and Supplementary Figure 3), which includes the cuboctahedron {NbCu_12_O_8_} cage-cluster via sharing Cu atoms (Figure 1E). It is noteworthy that this cuboctahedron-in-octahedron structure is unusual in heteropolyniobates but polyniobates with a rich variety of structures have been reported.

The third shell is a regular cubic cage-cluster {Cu_12_(Nb_7_(OH)O_21_)_8_} contains eight {Nb_7_(OH)O_21_}^8−^ cluster units, which cover on triangle faces of the shell **2** {Cu_12_I_3_O_3_} octahedron. Every {Nb_7_(OH)O_21_}^8−^ cluster unit captures three Cu atoms by three Cu-O-Nb bonds (Figure 1E). The 12 four-coordinated Cu atoms bridge the {Nb_7_(OH)O_21_}^8−^ cluster units to generate a regular cubic cage (Figure 1F and Supplementary Figure 4). Every {Nb_7_(OH)O_21_}^8−^ polyanion is located at each of its eight vertexes while the four-coordinated Cu atoms are at the edge of the cube. The 12 four-coordinated CuO_4_ all adopt square geometries: four O atoms from two {Nb_7_(OH)O_21_}^8−^ cluster units (Supplementary Figure 5). At the same time, each four-coordinated Cu atom is connected to one six-coordinated Cu atom by two bridged O atoms (Supplementary Figure 5).

**Figure 2:**
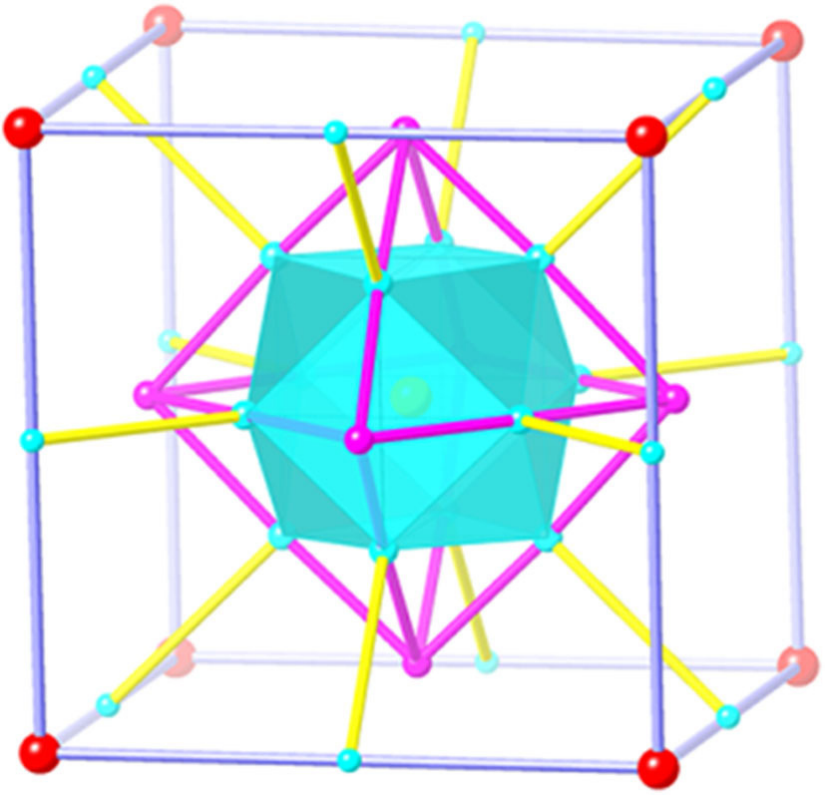
The topology of three-shell {(MCu_12_O_8_)(Cu_12_X_m_O_n_)(Nb_7_(OH)O_21_)_8_} polyanion [M = Nb (**1**), Nb (**2**), Ln (**3**); X = I (**1**, *m* = 3, *n* = 3; **2**, *m* = 5, *n* = 1), Br (**3**, *m* = 5, *n* = 1)] in compound **1**, **2**, and **3**. M, yellow ball; Cu, cyan ball; X/O, purple ball, [Nb_7_(OH)O_21_]_8_: red ball.

The most striking feature of **1** is the link between four-coordinated Cu atoms among the polyoxoniobate clusters and cupric clusters, which creates a cuboctahedron-in-octahedron-in-cube heteropolyoxo niobates (Figure 2). Notably, the structure of compound **1** is related to that of the polyanion {Cu_25.5_O_8_(Nb_7_O_22_)_8_} reported by Niu et al. (2007). They both contain {MCu_12_O_8_} and have cube-shaped {Cu_12_ (Nb_7_O_22_)_8_} clusters. However, there are three differences between these structures: (1) in compound **1**, the cube-shaped {Cu_12_(Nb_7_O_22_)_8_} cluster encapsulates an octahedral {Cu_12_I_3_O_3_} cluster, which was not found in {Cu_25.5_O_8_(Nb_7_O_22_)_8_}. Thus, compound **1** presents a new type of unusual structure of polyoxoniobates; (2) **1** captures one Nb atom in the central position, while there are Cu^2+^ atoms in {Cu_25.5_O_8_(Nb_7_O_22_)_8_} (Niu et al., 2007); and (3) in **1**, there is a protonated oxygen atom on every {Nb_7_(OH)O_21_} cluster unit, seen in Supplementary Tables 3–5 and Supplementary Figure 4, resulting in a slightly longer bond length of Nb-O, ranging from 1.859 (4)−2.472 (4) Å.

The heteropolyoxoniobate clusters {(NbO_8_Cu_24_I_3_O_3_)(Nb_7_(OH)O_21_)_8_}^36−^ are also connected each other by alkali Na^+^ cations that form a 3D inorganic polyoxometalate framework that exhibits different channels, which are filled with alkali metal cations and neutral guest molecules (Supplementary Figure 6).

Compound **2** reveals another giant cube-shaped cupreous-Iodide incorporated polyniobate, {(NbO_8_Cu_24_I_5_O)(Nb_7_(OH)-O_21_)_8_}^34−^, which is similar to the {(NbO_8_Cu_24_I_3_O_3_)(Nb_7_(OH)O_21_)_8_}^36−^ polyanion of **1** (Supplementary Figures 7, 8), except for the {Cu_12_I_5_O} octahedron in the second shell. In compound **2**, the second shell is an octahedral {Cu_12_I_5_O} cage-cluster with six disordered iodine/oxygen bridges. Each iodine/oxygen site is shared by iodine and oxygen atoms simultaneously. The site occupancies of the I_1_, I_1_' (1-x, 1-y, −4-z), I_2_, I_2_' (1-x, 1-y, −4-z), I_3_, I_3_' (1-x, 1-y, −4-z) anions are 0.80, 0.80, 0.85, 0.85, 0.85, and 0.85, respectively. The shared site occupancies of O_1M_, O_1M_' (1-x, 1-y, −4-z), O_2M_, O_2M_' (1-x, 1-y, −4-z), O_3M_, O_3M_' (1-x, 1-y, −4-z) anions are 0.20, 0.20, 0.15, 0.15, 0.15, and 0.15, respectively. It consists of five I atoms and one O atom in the {Cu_12_I_5_O} cage-cluster of **2** and comprises three I atoms and three O atoms in the {Cu_12_I_3_O_3_} cage-cluster of **1**. All copper atoms are +2 in compound **2**, as verified by BVS and XPS analysis (XPS, Supplementary Figure 2). In the packing diagram, polyoxoniobates {(NbO_8_Cu_24_I_5_O)(-Nb_7_(OH)O_21_)_8_}^34−^ are arranged in parallel along the *a, b*, and *c* axes, with {Na_4_(H_2_O)_19_} clusters as charge compensating cations and free water molecules, filling the gaps between polyoxoanions (Supplementary Figures 9, 10).

It is possible to effectively vary the central core metal atoms and the halogen atoms to obtain compound **3**. It shows a cube-shaped cupreous-bromide incorporated polyoxoniobate, {(LnO_8_Cu_24_Br_5_O)(Nb_7_(OH)O_21_)_8_}^36−^ similar to the {(NbO_8_Cu_24_I_3_O_3_)(Nb_7_(OH)-O_21_)_8_}^36−^ polyanion of **1**. The structural differences of compounds **1** and **3** are due to the fact that the Br^−^ replaces I^−^ while the {LnO_8_} cores supplant the {NbO_8_} cube-like unit with bond lengths of Cu-Br ranging from 3.054 (7) to 3.137 (6) Å. In the {LnO_8_} core, the central Ln atom is disordered at seven positions, and the site occupancies of the Gd_1_, Gd_2A_, Gd_3A_, Gd_4A_, Gd_5A_, Gd_6A_, and Gd_7A_ atoms are 0.670, 0.030, 0.075, 0.070, 0.065, 0.060, and 0.030, respectively. There is, therefore, one Gd atom in the central core. The lanthanide (Gd, Eu, Tb, Dy, La, and Nd) cations are first found in the center of polyoxoniobate (XPS, Supplementary Figures 11, 12; EDS-Mapping, Supplementary Figures 26, 27). Two {LnO_8_Cu_24_Br_5_O)(Nb_7_(OH)O_21_)_8_} clusters are bridged by two Na^+^ to form a polyanion dimer. The polyanion dimers are arranged in parallel along the *a, b*, and *c* axes (Supplementary Figure 13).

## Based-Catalysis Properties

Previous investigations indicate that PONbs can catalyze the hydrolytic decomposition of chemical warfare agent simulants, such as dimethyl methylphosphonate (DMMP) and diethyl cyanophosphonate (DECP) (Guo et al., 2016). Taking this into account, we tested the catalytic performance of **1**, **2**, and **3** in the hydrolytic decontamination of the nerve agent simulants DMMP and DECP.

Because purity and stability are important in the viability of a catalyst, we conducted experiments on the purity and stability of **1**, **2** and **3** (Supplementary Figures 14–27). Compounds **1**, **2**, and **3** were immersed in water and recollected for IR spectra. The IR spectra of **1**, **2**, and **3** after immersion were consistent with those before. This indicates that compounds **1**, **2**, and **3** maintain physical integrity and that no other new phases were generated (Supplementary Figures 28–30).

**Figure 3:**
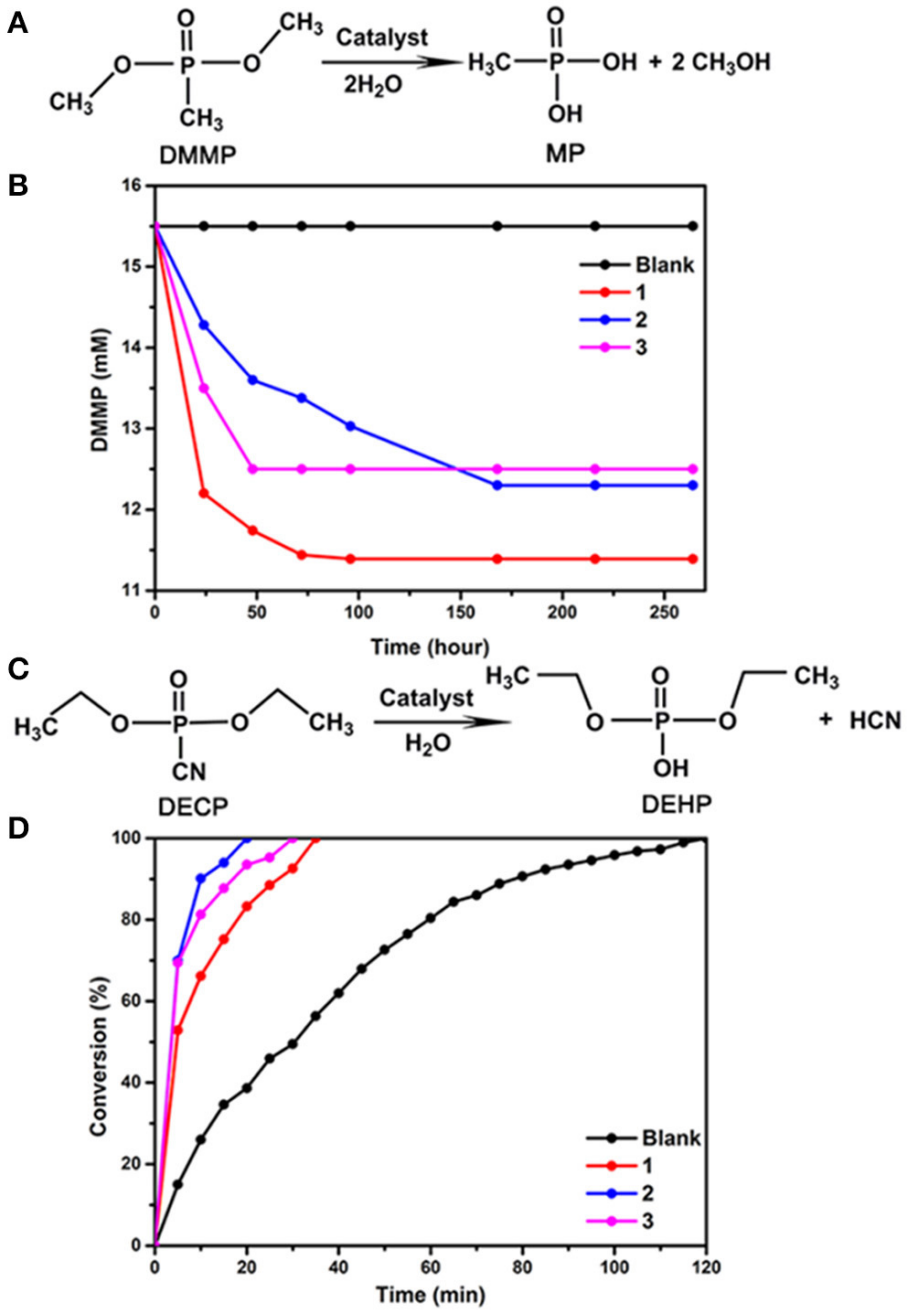
**(A)** Hydrolytic decomposition of DMMP to MP using catalyst **1** or **2** or **3**, Reaction conditions: DMMP (15.5 mM, 2.5 μL), catalyst **1** or **2** or **3** (50 mg), D_2_O (0.6 mL), and H_2_O (1.0 mL) at room temperature, **(B)** temporal course of the degradation of DMMP without catalyst and in aqueous dispersions of catalysts **1**, **2**, and **3**, **(C)** hydrolytic decontamination of DECP to DEHP using catalysts **1**, or **2** or **3**, Reaction conditions: DECP (100 mM, 10 μL), catalyst **1** or **2** or **3** (30 mg), DMF (600 μL) of and H_2_O (50 μL) at room temperature, **(D)** conversion of DECP to DEHP vs. reaction time without catalyst and using catalysts **1**, **2**, and **3**.

To evaluate the catalytic properties of compounds **1**, **2** and **3** for CWA destruction, we first analyzed the hydrolysis of dimethyl 4-nitrophenyl phosphate (DMMP). Fifty milligram samples **1**, **2**, or **3** were used as catalysts, and 15.5 mM of DMMP was dispersed in 1 mL of H_2_O and 0.6 mL D_2_O at room temperature and 1 atm. The results showed that 26.5, 20.63, and 19.35% DMMP was converted to methyl phosphoric acid in 264 h when compounds **1**, **2**, and **3** were used, respectively (Figure 3 and Supplementary Figures 31–33). In contrast, no non-toxic degradation production methyl phosphonic acid (MP) of DMMP can be detected in the absence of **1**, **2**, and **3**, suggesting that all of them are efficient DMMP catalysts. Their hydrolytic reactivity is weaker than that of KGeNb (54% conversion under the same reaction conditions) (Guo et al., 2016). Compound **1** is more active than **2** and **3** relatively. The IR spectra of compounds **1**, **2**, and **3** after catalytic hydrolysis were consistent with those before catalytic reaction, respectively, indicating that the structures of compounds **1**, **2**, and **3** remain unchanged (Supplementary Figures 34–36).

We further investigated the catalytic performance of compounds **1**, **2**, and **3** in the hydrolytic degradation of another chemical warfare agent, DECP to diethyl hydrogen phosphate (DEHP). As shown in Figure 3, 100% of DECP was converted by **1** in 35 min, by **2** in 20 min, and by **3** in 30 min, respectively. The hydrolysis of the P-CN bond was monitored by ^31^P NMR spectroscopy. The results reveal that compounds **1**, **2**, and **3** can greatly accelerate the hydrolytic reaction. These catalytic activities are comparable to that of KGeNb (Guo et al., 2016) reported by Hill and co-workers.

The reusability of compounds **1**, **2**, and **3** was also evaluated in the degradation of DECP (Supplementary Figures 37–39). The catalytic activities of compounds **1**, **2**, and **3** were maintained after 3 cycles. The IR spectra and XRD patterns of compounds **1**, **2**, and **3** after the three-cycle catalytic degradation of DECP reveal their crystalline integrity in catalytic reaction (Supplementary Figures 40–45). Compared with the decomposition of DMMP, the complete hydrolytic degradation of DECP under mild ambient conditions is of interest and has practical applications in providing human protection in real-world environments. Compounds **1**, **2**, and **3** contain basic {(MCu_12_O_8_)(Cu_12_X_m_O_n_)(Nb_7_O_22_)_8_} clusters with high negative charges. The protonation of the {(MO_8_Cu_24_X_m_O_n_)(Nb_7_(OH)-O_21_)_8_} could be the key step of the overall mechanism (Wang et al., 2017). The incorporation of X into the cluster is favorable for the hydrolytic degradation of chemical warfare agents.

## Conclusions

The first series of cube-shaped CuX-incorporated polyoxoniobates **1**, **2**, and **3** have been constructed based on the {MCu_12_O_8_} cluster, the {Cu_12_X_m_O_n_} cluster, and the {Nb_7_(OH)O_21_}^8−^ subunits under hydrothermal conditions. The cuboctahedron-in-octahedron-in-cube structure of {(MO_8_Cu_24_X_m_O_n_)(Nb_7_(OH)O_21_)_8_} polyanion is a new structure of CuX-incorporated heteropolyniobates. Compounds **1**, **2**, and **3** can effectively catalyze the hydrolytic degradation of the nerve agent simulants DECP (conv. 100% in 20–35 min) and DMMP. The incorporation of a cupreous-halid cluster into PONbs not only enriches the limited structural type of PONbs but also improves the hydrolytic activities.

## Data Availability Statement

The original contributions presented in the study are included in the article/Supplementary Materials, further inquiries can be directed to the corresponding author/s.

## Author Contributions

All experimental work was performed by Y-LW under the guidance of S-TZ, Y-QS, and X-XL. The manuscript was written by Y-LW with contributions and corrections from Y-QS and S-TZ. All authors contributed to the article and approved the submitted version.

## Conflict of Interest

The authors declare that the research was conducted in the absence of any commercial or financial relationships that could be construed as a potential conflict of interest.

## References

[B1] Bonhomme F. Larentzos J. P. Alam T. M. Maginn E. J. Nyman M. (2005). Synthesis, structural characterization, and molecular modeling of dodecaniobate keggin chain materials. Inorg. Chem. 44, 1774–1785. 10.1021/ic048847+ 15762704

[B2] Bontchev R. P. Nyman M. (2006). Evolution of polyoxoniobate cluster anions. Angew. Chem. Int. Ed. 45, 6670–6672. 10.1002/anie.200602200 16986204

[B3] Dong J. Hu J. F. Chi Y. N. Lin Z. G. Zou B. Yang S.. (2017). A polyoxoniobate-polyoxovanadate double-anion catalyst for simultaneous oxidative and hydrolytic decontamination of chemical warfare agent simulants. Angew. Chem. Int. Ed. 56, 4473–4477. 10.1002/anie.201700159 28322483

[B4] Guo W. W. Lv H. J. Sullivan K. P. Gordon W. O. Balboa A. Wagner G. W.. (2016). Broad-spectrum liquid-and gas-phase decontamination of chemical warfare agents by one-dimensional heteropolyniobates. Angew. Chem. Int. Ed. 55, 7403–7407. 10.1002/anie.201601620 27061963

[B5] Guo Z. W. Chen Y. Zhao D. Wu Y. L. Lin L. D. Zheng S. T. (2019). A chromium-substituted polyoxoniobate with high ionic conductivity. Inorg. Chem. 58, 4055–4058. 10.1021/acs.inorgchem.8b03529 30868872

[B6] Hu J. F. Dong J. Huang X. Q. Chi Y. N. Lin Z. G. Li J. K.. (2017). Immobilization of Keggin polyoxovanadoniobate in crystalline solids to produce effective heterogeneous catalysts towards selective oxidation of benzyl-alkanes. Dalton Trans. 46, 8245–8251. 10.1039/C7DT01122A 28612860

[B7] Hu J. F. Han T. Chi Y. N. Lin Z. G. Xu Y. Q. Yang S.. (2016). Sulfur-centred polyoxoniobate-based 3D organic-inorganic hybrid compound and its magnetic behavior. Chem. Commun. 52, 10846–10849. 10.1039/C6CC03915D 27430194

[B8] Huang P. Qin C. Su Z. M. Xing Y. Wang X. L. Shao K. Z.. (2012a). Self-assembly and photocatalytic properties of polyoxoniobates: {Nb_24_O_72_}, {Nb_32_O_96_}, and {K_12_Nb_96_O_288_} clusters. J. Am. Chem. Soc. 134, 14004–14010. 10.1021/ja303723u 22905866

[B9] Huang P. Qin C. Wang X. L. Sun C. Y. Yang G. S. Shao K. Z.. (2012b). An unprecedented organic-inorganic hybrid based on the first {Nb_10_V_4_O_40_(OH)_2_}^12−^clusters and copper cations. Chem. Commun. 48, 103–105. 10.1039/C1CC15684E 22057477

[B10] Ivanchikova I. D. Maksimchuk N. V. Maksimovskaya R. I. Maksimov G. M. Kholdeeva O. A. (2014). Highly selective oxidation of alkylphenols to *p*-benzoquinones with aqueous hydrogen peroxide catalyzed by divanadium-substituted polyoxotungstates. ACS Catal. 4, 2706–2713. 10.1021/cs500738e

[B11] Jin L. Li X. X. Qi Y. J. Niu P. P. Zheng S. T. (2016). Giant hollow heterometallic polyoxoniobates with sodalite-type lanthanide-tungsten-oxide cages: discrete nanoclusters and extended frameworks. Angew. Chem. Int. Ed. 55, 13793–13797. 10.1002/anie.201608113 27678257

[B12] Jin L. Zhu Z. K. Wu Y. L. Qi Y. J. Li X. X. Zheng S. T. (2017). Record high-nuclearity polyoxoniobates: discrete nanoclusters {nb_114_}, {nb_81_}, and {nb_52_}, and extended frameworks based on {Cu_3_Nb_78_} and {Cu_4_Nb_78_}. Angew. Chem. Int. Ed. 56, 16288–16292. 10.1002/anie.201709565 29105960

[B13] Judd D. A. Nettles J. H. Nevins N. Snyder J. P. Liotta D. C. Tang J.. (2001). Polyoxometalate HIV-1 protease inhibitors. A new mode of protease inhibition. J. Am. Chem. Soc. 123, 886–897. 10.1021/ja001809e 11456622

[B14] Kim T. H. Shin Y. W. Jung J. H. Kim J. S. Kim J. (2008). Crystal–to-crystal transformation between three cu^i^ coordination polymers and structural evidence for luminescence thermochromism. Angew. Chem. Int. Ed. 47, 685–688. 10.1002/anie.200704349 18080268

[B15] Lee J. Y. Lee S. Y. Sim W. Park K. M. Kim J. Lee S. S. (2008). Temperature-dependent 3-D cui coordination polymers of calix^4^-*BIS*-dithiacrown: crystal-to-crystal transformation and photoluminescence change on coordinated solvent removal. J. Am. Chem. Soc. 130, 6902–6903. 10.1021/ja8008693 18470989

[B16] Liang Z. J. Qiao Y. Y. Li M. M. Ma P. T. Niu J. Y. Wang J. P. (2019). Two synthetic routes generate two isopolyoxoniobates based on {Nb_16_} and {Nb_20_}. Dalton Trans. 48, 17709–17712. 10.1039/C9DT04147H 31777893

[B17] Liang Z. J. Wang K. Zhang D. D. Ma P. T. Niu J. Y. Wang J. P. (2017). {Fe_3_Nb_25_} cluster based on an Fe-centred Keggin unit. Dalton Trans. 46, 1368–1371. 10.1039/C6DT04223F 28067387

[B18] López-Maya E. Montoro C. Rodríguez-Albelo L. M. Cervantes S. D. A. Lozano-Pérez A. A. Cenís J. L.. (2015). Textile/metal-organic-framework composites as self-detoxifying filters for chemical-warfare agents. Angew. Chem. Int. Ed. 54, 6790–6794. 10.1002/anie.201502094 25951010

[B19] Maekaw M. Ozawa Y. Yagasaki A. (2006). Icosaniobate: a new member of the isoniobate family. Inorg. Chem. 45, 9608–9926. 10.1021/ic0601788 17112246

[B20] Niu J. Y. Li F. Zhao J. W. Ma P. T. Zhang D. D. Bassil B.. (2014). Tetradecacobalt(II)-Containing 36-niobate [Co_14_(OH)_16_(H_2_O)_8_Nb_36_O_106_]^20−^ and its photocatalytic H_2_ evolution activity. Chem. Eur. J. 20, 9852–9857. 10.1002/chem.201402730 25043175

[B21] Niu J. Y. Ma P. T. Niu H. Y. Li J. Zhao J. W. Song Y.. (2007). Giant polyniobate clusters based on [Nb_7_O_22_]^9−^ units derived from a Nb_6_O_19_ precursor. Chem. Eur. J. 13, 8739–8748. 10.1002/chem.200700612 17654454

[B22] Nyman M. Bonhomme F. Alam T. M. Rodriguez M. A. Cherry B. R. Krumhansl J. L.. (2002). A general synthetic procedure for heteropolyniobates. Science 297, 996–998. 10.1126/science.1073979 12169730

[B23] Ohlin C. A. Villa E. M. Fettinger J. C. Casey W. H. (2008). The [Ti_12_Nb_6_O_44_]^10−^ ion-a new type of polyoxometalate structure. Angew. Chem. Int. Ed. 47, 5634–5636. 10.1002/anie.200801883 18567045

[B24] Pantenburg I. Müller I. (2004). Ein netzwerk aus iodid-ionen und iod-molekülen in der kristallstruktur von [Pr(Benzo-15-Krone-5)_2_]I_21_. Z. Anorg, Allg. Chem. 630, 1637–1640. 10.1002/zaac.200400199

[B25] Qiao X. Q. Zhang Z. W. Li Q. H. Hou D. F. Zhang Q. C. Zhang J. (2018). In situ synthesis of n-n Bi_2_MoO_6_ & Bi_2_S_3_ heterojunctions for highly efficient photocatalytic removal of Cr (vi). J. Mater. Chem. A 6, 22580–22589. 10.1039/C8TA08294D

[B26] Shan X. C. Jiang F. L. Yuan D. Q. Zhang H. B. Wu M. Y. Chen L. (2013). A multi-metal-cluster MOF with Cu_4_I_4_ and Cu_6_S_6_ as functional groups exhibiting dual emission with both thermochromic and near-IR character. Chem. Sci. 4, 1484–1489. 10.1039/c3sc21995j

[B27] Shen L. Li C. H. Chi Y. N. Hu C. W. (2008). Zn(2,2′-bipy)_2_/Co(2,2′-bipy)_2_ linked decaniobate [Nb_10_O_28_]^6−^ clusters–zigzag neutral chains. Inorg. Chem. Commun. 11, 992–994. 10.1016/j.inoche.2008.05.015

[B28] Tsunashima R. Long D. L. Miras H. N. Gabb D. Pradeep C. P. Cronin L. (2010). The Construction of high-nuclearity isopolyoxoniobates with pentagonal building blocks:[HNb_27_O_76_]^16−^ and [H_10_Nb_31_O_93_(CO_3_)]^23−^. Angew. Chem. Int. Ed. 49, 113–116. 10.1002/anie.200903970 19946917

[B29] Wang Q. R. C. Jr Plonka A. M. Gordon W. O. Guo W. W. Nguyen-Phan T. D. Sharp C. H.. (2017). Atomic-level structural dynamics of polyoxoniobates during DMMP decomposition. Sci. Rep. 773, 1–7. 10.1038/s41598-017-00772-x 28396583 PMC5429595

[B30] Wang Z. Sun H. T. Kurmoo M. Liu Q. Y. Zhuang G. L. Zhao Q. Q.. (2019). Carboxylic acid stimulated silver shell isomerism in a triple core–shell Ag_84_ nanocluster. Chem. Sci. 10, 4862–4867. 10.1039/C8SC05666H 31183036 PMC6520922

[B31] Wu Y. L. Li X. X. Qi Y. J. Yu H. Jin L. Zheng S. T. (2018). {Nb_288_O_768_(OH)_48_(CO_3_)_12_}: A macromolecular polyoxometalate with close to 300 niobium atoms. Angew. Chem. Int. Ed. 57, 8572–8576. 10.1002/anie.201804088 29809317

[B32] Yu Y. Zhang X. M. Ma J. P. Liu Q. K. Wang P. Dong Y. B. (2014). Cu(i)-MOF: naked-eye colorimetric sensor for humidity and formaldehyde in single-crystal-to-single-crystal fashion. Chem. Commun. 50, 1444–1446. 10.1039/C3CC47723A 24352589

[B33] Zhang Z. Y. Lin Q. P. Kurunthu D. Wu T. Zuo F. Zheng S. T.. (2011). Synthesis and photocatalytic properties of a new heteropolyoxoniobate compound: K_10_[Nb_2_O_2_(H_2_O)_2_][SiNb_12_O_40_]·12H_2_O. J. Am. Chem. Soc. 133, 6934–6937. 10.1021/ja201670x 21500805

[B34] Zhao J. J. Lee D. T. Yaga R. W. Hall M. G. Barton H. F. Woodward I. R.. (2016). Ultra-fast degradation of chemical warfare agents using MOF-nanofiber kebabs. Angew. Chem. Int. Ed. 128, 13418–13422. 10.1002/ange.201606656 27653957

[B35] Zhao M. J. Chen S. M. Huang Y. T. Dan Y. M. (2017). An unusual 2p-3d-4f heterometallic coordination polymer featuring Ln_8_Na and Cu_8_I clusters as nodes. J. Mol. Struct. 1128, 123–126. 10.1016/j.molstruc.2016.08.027

[B36] Zhu H. J. Lu M. Wang Y. R. Yao S. J. Zhang M. Kan Y. H.. (2020). Efficient electron transmission in covalent organic framework nanosheets for highly active electrocatalytic carbon dioxide reduction. Nat. Commun. 11:497. 10.1038/s41467-019-14237-4 31980641 PMC6981265

