# Supplementary material for: A Series of Cube-Shaped Polyoxoniobates Encapsulating Octahedral Cu12XmOn Clusters With Hydrolytic Decomposition for Chemical Warfare Agents
Source: Front Chem. 2020 Dec 18;8:586009. doi: 10.3389/fchem.2020.586009 (PMC7775552; doi:10.3389/fchem.2020.586009)

checkCIF/PLATON report

Structure factors have been supplied for datablock(s) 1

THIS REPORT IS FOR GUIDANCE ONLY. IF USED AS PART OF A REVIEW PROCEDURE FOR PUBLICATION, IT SHOULD NOT REPLACE THE EXPERTISE OF AN EXPERIENCED CRYSTALLOGRAPHIC REFEREE.

No syntax errors found. CIF dictionary Interpreting this report

Datablock: 1

```
Bond precision:  Cu- O = 0.0043 A        Wavelength=0.71073

Cell:          a=20.3063(14)      b=20.9947(13)     c=21.1179(14)
               alpha=80.0251(12)  beta=88.5830(11)  gamma=84.7731(11)
Temperature:  175 K
```

	Calculated	Reported
Volume	8829.8(10)	8829.8(10)
Space group	P -1	P -1
Hall group	-P 1	-P 1
Moiety formula	Cu24 I3 Nb57 O189, 32(O), 12(Na) [+ solvent]	?
Sum formula	Cu24 I3 Na12 Nb57 O221 [+ solvent]	Cu24 I3 Na12 Nb57 O221
Mr	11013.60	11013.41
Dx,g cm-3	2.071	2.071
Z	1	1
Mu (mm-1)	3.530	3.530
F000	5092.0	5092.0
F000’	4980.55	
h,k,lmax	24,24,25	24,24,25
Nref	31085	30673
Tmin,Tmax	0.616,0.655	0.697,0.745
Tmin’	0.604	

```
Correction method= # Reported T Limits: Tmin=0.697 Tmax=0.745
AbsCorr = NONE

Data completeness= 0.987           Theta(max)= 24.998

R(reflections)= 0.0436( 26357)    wR2(reflections)= 0.1465( 30673)

S = 1.015                   Npar= 1463
```

The following ALERTS were generated. Each ALERT has the format
test-name_ALERT_alert-type_alert-level.
Click on the hyperlinks for more details of the test.

Alert level B

```
PLAT220_ALERT_2_B NonSolvent Resd 1  O   Ueq(max) / Ueq(min) Range        7.7 Ratio
PLAT306_ALERT_2_B Isolated Oxygen Atom (H-atoms Missing ?) .......        O1W Check
PLAT306_ALERT_2_B Isolated Oxygen Atom (H-atoms Missing ?) .......        O2W Check
PLAT306_ALERT_2_B Isolated Oxygen Atom (H-atoms Missing ?) .......        O3W Check
PLAT306_ALERT_2_B Isolated Oxygen Atom (H-atoms Missing ?) .......        O6W Check
PLAT306_ALERT_2_B Isolated Oxygen Atom (H-atoms Missing ?) .......        O7W Check
PLAT306_ALERT_2_B Isolated Oxygen Atom (H-atoms Missing ?) .......        O8W Check
PLAT306_ALERT_2_B Isolated Oxygen Atom (H-atoms Missing ?) .......        O9W Check
PLAT306_ALERT_2_B Isolated Oxygen Atom (H-atoms Missing ?) .......       O10W Check
PLAT306_ALERT_2_B Isolated Oxygen Atom (H-atoms Missing ?) .......       O12W Check
PLAT306_ALERT_2_B Isolated Oxygen Atom (H-atoms Missing ?) .......       O13W Check
PLAT306_ALERT_2_B Isolated Oxygen Atom (H-atoms Missing ?) .......       O15W Check
PLAT306_ALERT_2_B Isolated Oxygen Atom (H-atoms Missing ?) .......       O16W Check
PLAT306_ALERT_2_B Isolated Oxygen Atom (H-atoms Missing ?) .......       O17W Check
PLAT306_ALERT_2_B Isolated Oxygen Atom (H-atoms Missing ?) .......       O18W Check
PLAT306_ALERT_2_B Isolated Oxygen Atom (H-atoms Missing ?) .......       O25W Check
PLAT975_ALERT_2_B Check Calcd Resid. Dens.  1.03A   From O10W          1.84 eA-3
PLAT990_ALERT_1_B Deprecated .res/.hkl Input Style SQUEEZE Job ...          ! Note
```

Alert level C

```
DIFMX02_ALERT_1_C  The maximum difference density is > 0.1*ZMAX*0.75
            The relevant atom site should be identified.
PLAT018_ALERT_1_C _diffrn_measured_fraction_theta_max .NE. *_full           ! Check
PLAT094_ALERT_2_C Ratio of Maximum / Minimum Residual Density ....       2.17 Report
PLAT097_ALERT_2_C Large Reported Max.  (Positive) Residual Density       4.67 eA-3
PLAT220_ALERT_2_C NonSolvent Resd 1 Nb   Ueq(max) / Ueq(min) Range        3.5 Ratio
PLAT241_ALERT_2_C High   'MainMol' Ueq as Compared to Neighbors of         O4 Check
PLAT242_ALERT_2_C Low    'MainMol' Ueq as Compared to Neighbors of        Cu3 Check
PLAT242_ALERT_2_C Low    'MainMol' Ueq as Compared to Neighbors of       Cu16 Check
PLAT260_ALERT_2_C Large Average Ueq of Residue Including      O17W      0.102 Check
PLAT790_ALERT_4_C Centre of Gravity not Within Unit Cell: Resd.  #          1 Note
              Cu24 I3 Nb57 O189
PLAT910_ALERT_3_C Missing # of FCF Reflection(s) Below Theta(Min).          6 Note
PLAT911_ALERT_3_C Missing FCF Refl Between Thmin & STh/L=    0.595        406 Report
PLAT918_ALERT_3_C Reflection(s) with I(obs) much Smaller I(calc) .          3 Check
PLAT934_ALERT_3_C Number of (Iobs-Icalc)/Sigma(W) > 10 Outliers ..          1 Check
PLAT976_ALERT_2_C Check Calcd Resid. Dens.  0.42A   From O17W           -0.91 eA-3
PLAT976_ALERT_2_C Check Calcd Resid. Dens.  0.77A   From O91            -0.74 eA-3
```

Alert level G

```
PLAT003_ALERT_2_G Number of Uiso or Uij Restrained non-H Atoms ...        160 Report
PLAT072_ALERT_2_G SHELXL First  Parameter in WGHT  Unusually Large       0.11 Report
PLAT083_ALERT_2_G SHELXL Second Parameter in WGHT  Unusually Large      33.00 Why ?
PLAT168_ALERT_4_G The CIF-Embedded .res File Contains EXYZ Records          3 Report
PLAT171_ALERT_4_G The CIF-Embedded .res File Contains EADP Records          3 Report
PLAT186_ALERT_4_G The CIF-Embedded .res File Contains ISOR Records          1 Report
PLAT187_ALERT_4_G The CIF-Embedded .res File Contains RIGU Records          1 Report
PLAT300_ALERT_4_G Atom Site Occupancy of I1         Constrained at       0.55 Check
PLAT300_ALERT_4_G Atom Site Occupancy of I2         Constrained at        0.5 Check
PLAT300_ALERT_4_G Atom Site Occupancy of I3         Constrained at       0.45 Check
PLAT300_ALERT_4_G Atom Site Occupancy of O2M        Constrained at        0.5 Check
PLAT300_ALERT_4_G Atom Site Occupancy of O3M        Constrained at       0.55 Check
PLAT300_ALERT_4_G Atom Site Occupancy of O1M        Constrained at       0.45 Check
```

```
PLAT301_ALERT_3_G Main Residue  Disorder .............(Resd  1  )         2% Note
PLAT302_ALERT_4_G Anion/Solvent/Minor-Residue Disorder (Resd  4  )       100% Note
PLAT302_ALERT_4_G Anion/Solvent/Minor-Residue Disorder (Resd 18  )       100% Note
PLAT304_ALERT_4_G Non-Integer Number of Atoms in ..... (Resd  4  )       0.55 Check
PLAT304_ALERT_4_G Non-Integer Number of Atoms in ..... (Resd 18  )       0.45 Check
PLAT311_ALERT_2_G Isolated Disordered Oxygen Atom (No H's ?) .....        O2M Check
PLAT311_ALERT_2_G Isolated Disordered Oxygen Atom (No H's ?) .....        O3M Check
PLAT311_ALERT_2_G Isolated Disordered Oxygen Atom (No H's ?) .....        O1M Check
PLAT606_ALERT_4_G VERY LARGE Solvent Accessible VOID(S) in Structure        ! Info
PLAT789_ALERT_4_G Atoms with Negative _atom_site_disorder_group  #          3 Check
PLAT790_ALERT_4_G Centre of Gravity not Within Unit Cell: Resd.  #          2 Note
              O
PLAT790_ALERT_4_G Centre of Gravity not Within Unit Cell: Resd.  #          3 Note
              O
PLAT790_ALERT_4_G Centre of Gravity not Within Unit Cell: Resd.  #          4 Note
              O
PLAT790_ALERT_4_G Centre of Gravity not Within Unit Cell: Resd.  #          5 Note
              O
PLAT790_ALERT_4_G Centre of Gravity not Within Unit Cell: Resd.  #          6 Note
              O
PLAT790_ALERT_4_G Centre of Gravity not Within Unit Cell: Resd.  #          7 Note
              O
PLAT790_ALERT_4_G Centre of Gravity not Within Unit Cell: Resd.  #          8 Note
              O
PLAT790_ALERT_4_G Centre of Gravity not Within Unit Cell: Resd.  #          9 Note
              O
PLAT790_ALERT_4_G Centre of Gravity not Within Unit Cell: Resd.  #         10 Note
              O
PLAT790_ALERT_4_G Centre of Gravity not Within Unit Cell: Resd.  #         11 Note
              O
PLAT790_ALERT_4_G Centre of Gravity not Within Unit Cell: Resd.  #         12 Note
              O
PLAT790_ALERT_4_G Centre of Gravity not Within Unit Cell: Resd.  #         13 Note
              O
PLAT790_ALERT_4_G Centre of Gravity not Within Unit Cell: Resd.  #         14 Note
              O
PLAT790_ALERT_4_G Centre of Gravity not Within Unit Cell: Resd.  #         15 Note
              O
PLAT790_ALERT_4_G Centre of Gravity not Within Unit Cell: Resd.  #         16 Note
              O
PLAT790_ALERT_4_G Centre of Gravity not Within Unit Cell: Resd.  #         17 Note
              O
PLAT790_ALERT_4_G Centre of Gravity not Within Unit Cell: Resd.  #         18 Note
              O
PLAT790_ALERT_4_G Centre of Gravity not Within Unit Cell: Resd.  #         19 Note
              Na
PLAT790_ALERT_4_G Centre of Gravity not Within Unit Cell: Resd.  #         20 Note
              Na
PLAT790_ALERT_4_G Centre of Gravity not Within Unit Cell: Resd.  #         21 Note
              Na
PLAT790_ALERT_4_G Centre of Gravity not Within Unit Cell: Resd.  #         22 Note
              Na
PLAT790_ALERT_4_G Centre of Gravity not Within Unit Cell: Resd.  #         23 Note
              Na
PLAT790_ALERT_4_G Centre of Gravity not Within Unit Cell: Resd.  #         24 Note
              Na
PLAT794_ALERT_5_G Tentative Bond Valency for Nb1       (V)       .       4.82 Info
PLAT794_ALERT_5_G Tentative Bond Valency for Nb2       (V)       .       4.86 Info
PLAT794_ALERT_5_G Tentative Bond Valency for Nb3       (V)       .       4.81 Info
PLAT794_ALERT_5_G Tentative Bond Valency for Nb4       (V)       .       5.00 Info
PLAT794_ALERT_5_G Tentative Bond Valency for Nb5       (V)       .       4.97 Info
PLAT794_ALERT_5_G Tentative Bond Valency for Nb6       (V)       .       4.96 Info
```

```
PLAT794_ALERT_5_G Tentative Bond Valency for Nb7       (V)       .       4.93 Info
PLAT794_ALERT_5_G Tentative Bond Valency for Nb8       (V)       .       4.91 Info
PLAT794_ALERT_5_G Tentative Bond Valency for Nb9       (V)       .       4.93 Info
PLAT794_ALERT_5_G Tentative Bond Valency for Nb10      (V)       .       4.89 Info
PLAT794_ALERT_5_G Tentative Bond Valency for Nb11      (V)       .       4.89 Info
PLAT794_ALERT_5_G Tentative Bond Valency for Nb12      (V)       .       4.91 Info
PLAT794_ALERT_5_G Tentative Bond Valency for Nb13      (V)       .       4.99 Info
PLAT794_ALERT_5_G Tentative Bond Valency for Nb14      (V)       .       4.92 Info
PLAT794_ALERT_5_G Tentative Bond Valency for Nb15      (V)       .       4.96 Info
PLAT794_ALERT_5_G Tentative Bond Valency for Nb16      (V)       .       4.94 Info
PLAT794_ALERT_5_G Tentative Bond Valency for Nb17      (V)       .       4.91 Info
PLAT794_ALERT_5_G Tentative Bond Valency for Nb18      (V)       .       4.92 Info
PLAT794_ALERT_5_G Tentative Bond Valency for Nb19      (V)       .       4.96 Info
PLAT794_ALERT_5_G Tentative Bond Valency for Nb20      (V)       .       4.99 Info
PLAT794_ALERT_5_G Tentative Bond Valency for Nb21      (V)       .       5.00 Info
PLAT794_ALERT_5_G Tentative Bond Valency for Nb22      (V)       .       4.98 Info
PLAT794_ALERT_5_G Tentative Bond Valency for Nb23      (V)       .       4.90 Info
PLAT794_ALERT_5_G Tentative Bond Valency for Nb24      (V)       .       5.01 Info
PLAT794_ALERT_5_G Tentative Bond Valency for Nb26      (V)       .       4.97 Info
PLAT794_ALERT_5_G Tentative Bond Valency for Nb27      (V)       .       5.03 Info
PLAT794_ALERT_5_G Tentative Bond Valency for Nb29      (V)       .       4.96 Info
PLAT794_ALERT_5_G Tentative Bond Valency for Nb36      (V)       .       4.95 Info
PLAT794_ALERT_5_G Tentative Bond Valency for Cu16      (II)      .       2.07 Info
PLAT860_ALERT_3_G Number of Least-Squares Restraints .............       3652 Note
PLAT869_ALERT_4_G ALERTS Related to the Use of SQUEEZE Suppressed           ! Info
PLAT883_ALERT_1_G No Info/Value for _atom_sites_solution_primary .     Please Do !
PLAT909_ALERT_3_G Percentage of I>2sig(I) Data at Theta(Max) Still        77% Note
PLAT933_ALERT_2_G Number of OMIT Records in Embedded .res File ...         13 Note
PLAT941_ALERT_3_G Average HKL Measurement Multiplicity ...........        2.7 Low
PLAT961_ALERT_5_G Dataset Contains no Negative Intensities .......     Please Check
PLAT965_ALERT_2_G The SHELXL WEIGHT Optimisation has not Converged     Please Check
PLAT992_ALERT_5_G Repd & Actual _reflns_number_gt Values Differ by          2 Check
```

---

0 **ALERT level A** = Most likely a serious problem - resolve or explain
18 **ALERT level B** = A potentially serious problem, consider carefully
16 **ALERT level C** = Check. Ensure it is not caused by an omission or oversight
84 **ALERT level G** = General information/check it is not something unexpected

4 ALERT type 1 CIF construction/syntax error, inconsistent or missing data
34 ALERT type 2 Indicator that the structure model may be wrong or deficient
8 ALERT type 3 Indicator that the structure quality may be low
41 ALERT type 4 Improvement, methodology, query or suggestion
31 ALERT type 5 Informative message, check

It is advisable to attempt to resolve as many as possible of the alerts in all categories. Often the minor alerts point to easily fixed oversights, errors and omissions in your CIF or refinement strategy, so attention to these fine details can be worthwhile. In order to resolve some of the more serious problems it may be necessary to carry out additional measurements or structure refinements. However, the purpose of your study may justify the reported deviations and the more serious of these should normally be commented upon in the discussion or experimental section of a paper or in the "special_details" fields of the CIF. checkCIF was carefully designed to identify outliers and unusual parameters, but every test has its limitations and alerts that are not important in a particular case may appear. Conversely, the absence of alerts does not guarantee there are no aspects of the results needing attention. It is up to the individual to critically assess their own results and, if necessary, seek expert advice.

Publication of your CIF in IUCr journals

A basic structural check has been run on your CIF. These basic checks will be run on all CIFs submitted for publication in IUCr journals (*Acta Crystallographica*, *Journal of Applied Crystallography*, *Journal of Synchrotron Radiation*); however, if you intend to submit to *Acta Crystallographica Section C* or *E* or *IUCrData*, you should make sure that full publication checks are run on the final version of your CIF prior to submission.

Publication of your CIF in other journals

Please refer to the *Notes for Authors* of the relevant journal for any special instructions relating to CIF submission.

PLATON version of 04/06/2020; check.def file version of 02/06/2020

Datablock 1 - ellipsoid plot

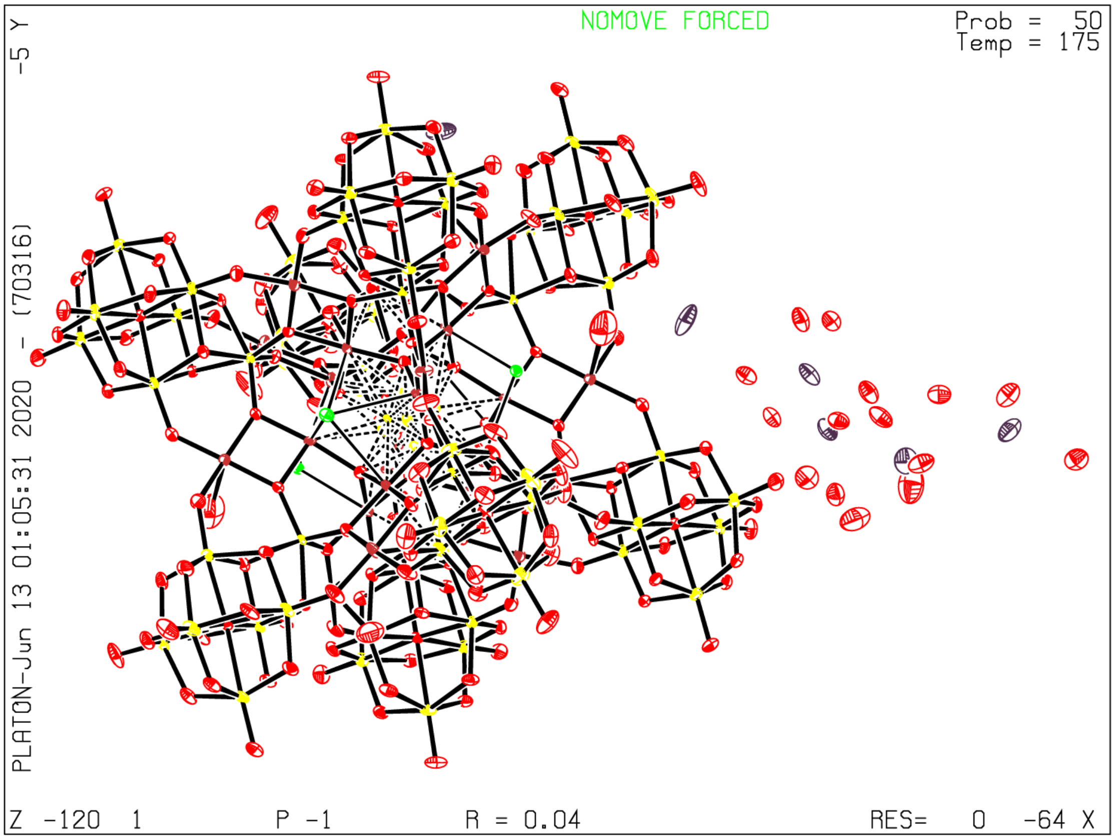

Supplement: Supplementary file 1 [file Data_Sheet_1.PDF]
