# Supplementary material for: A Series of Cube-Shaped Polyoxoniobates Encapsulating Octahedral Cu12XmOn Clusters With Hydrolytic Decomposition for Chemical Warfare Agents
Source: Front Chem. 2020 Dec 18;8:586009. doi: 10.3389/fchem.2020.586009 (PMC7775552; doi:10.3389/fchem.2020.586009)

# checkCIF/PLATON report

You have not supplied any structure factors. As a result the full set of tests cannot be run.

THIS REPORT IS FOR GUIDANCE ONLY. IF USED AS PART OF A REVIEW PROCEDURE FOR PUBLICATION, IT SHOULD NOT REPLACE THE EXPERTISE OF AN EXPERIENCED CRYSTALLOGRAPHIC REFEREE.

No syntax errors found. CIF dictionary Interpreting this report

## Datablock: 1

---

```
Bond precision:  Cu- O = 0.0041 A        Wavelength=0.71073

Cell:          a=20.2836(13)      b=21.0499(12)     c=21.0976(13)
               alpha=79.9971(10)  beta=88.5513(11)  gamma=84.6319(10)
Temperature:   175 K
```

| | Calculated | Reported |
|---|---|---|
| Volume | 8831.8(9) | 8831.8(9) |
| Space group | P -1 | P -1 |
| Hall group | -P 1 | -P 1 |
| Moiety formula | Cu24 I5 Nb57 O186, 55(O), 12(Na) [+ solvent] | ? |
| Sum formula | Cu24 I5 Na12 Nb57 O241 [+ solvent] | Cu24 I5 Na12 Nb57 O241 |
| Mr | 11587.42 | 11587.21 |
| Dx,g cm-3 | 2.179 | 2.179 |
| Z | 1 | 1 |
| Mu (mm-1) | 3.712 | 3.712 |
| F000 | 5358.0 | 5358.0 |
| F000’ | 5245.84 | |
| h,k,lmax | 24,25,25 | 24,25,25 |
| Nref | 31376 | 31085 |
| Tmin,Tmax | 0.558,0.595 | 0.209,0.259 |
| Tmin’ | 0.547 | |

```
Correction method= # Reported T Limits: Tmin=0.209 Tmax=0.259
AbsCorr = NONE

Data completeness= 0.991          Theta(max)= 25.076

R(reflections)= 0.0394( 26981)    wR2(reflections)= 0.1136( 31085)

S = 1.087                   Npar= 1542
```

---

```
The following ALERTS were generated. Each ALERT has the format
       test-name_ALERT_alert-type_alert-level.
Click on the hyperlinks for more details of the test.
```

---

### Alert level B

```
PLAT094_ALERT_2_B Ratio of Maximum / Minimum Residual Density ....       4.27 Report
PLAT306_ALERT_2_B Isolated Oxygen Atom (H-atoms Missing ?) .......        O4W Check
PLAT306_ALERT_2_B Isolated Oxygen Atom (H-atoms Missing ?) .......        O5W Check
PLAT306_ALERT_2_B Isolated Oxygen Atom (H-atoms Missing ?) .......        O6W Check
PLAT306_ALERT_2_B Isolated Oxygen Atom (H-atoms Missing ?) .......        O7W Check
PLAT306_ALERT_2_B Isolated Oxygen Atom (H-atoms Missing ?) .......        O9W Check
PLAT306_ALERT_2_B Isolated Oxygen Atom (H-atoms Missing ?) .......       O10W Check
PLAT306_ALERT_2_B Isolated Oxygen Atom (H-atoms Missing ?) .......       O11W Check
PLAT306_ALERT_2_B Isolated Oxygen Atom (H-atoms Missing ?) .......       O12W Check
PLAT306_ALERT_2_B Isolated Oxygen Atom (H-atoms Missing ?) .......       O13W Check
PLAT306_ALERT_2_B Isolated Oxygen Atom (H-atoms Missing ?) .......       O14W Check
PLAT306_ALERT_2_B Isolated Oxygen Atom (H-atoms Missing ?) .......       O15W Check
PLAT306_ALERT_2_B Isolated Oxygen Atom (H-atoms Missing ?) .......       O16W Check
PLAT306_ALERT_2_B Isolated Oxygen Atom (H-atoms Missing ?) .......       O17W Check
PLAT306_ALERT_2_B Isolated Oxygen Atom (H-atoms Missing ?) .......       O18W Check
PLAT306_ALERT_2_B Isolated Oxygen Atom (H-atoms Missing ?) .......       O19W Check
PLAT306_ALERT_2_B Isolated Oxygen Atom (H-atoms Missing ?) .......       O20W Check
PLAT306_ALERT_2_B Isolated Oxygen Atom (H-atoms Missing ?) .......       O21W Check
PLAT306_ALERT_2_B Isolated Oxygen Atom (H-atoms Missing ?) .......       O22W Check
PLAT306_ALERT_2_B Isolated Oxygen Atom (H-atoms Missing ?) .......       O23W Check
PLAT306_ALERT_2_B Isolated Oxygen Atom (H-atoms Missing ?) .......       O24W Check
PLAT306_ALERT_2_B Isolated Oxygen Atom (H-atoms Missing ?) .......       O25W Check
PLAT306_ALERT_2_B Isolated Oxygen Atom (H-atoms Missing ?) .......       O30W Check
PLAT306_ALERT_2_B Isolated Oxygen Atom (H-atoms Missing ?) .......       O34W Check
PLAT306_ALERT_2_B Isolated Oxygen Atom (H-atoms Missing ?) .......        O41 Check
PLAT306_ALERT_2_B Isolated Oxygen Atom (H-atoms Missing ?) .......        O48 Check
PLAT306_ALERT_2_B Isolated Oxygen Atom (H-atoms Missing ?) .......        O61 Check
PLAT306_ALERT_2_B Isolated Oxygen Atom (H-atoms Missing ?) .......        O94 Check
PLAT990_ALERT_1_B Deprecated .res/.hkl Input Style SQUEEZE Job ...          ! Note
```

---

### Alert level C

```
DIFMX02_ALERT_1_C  The maximum difference density is > 0.1*ZMAX*0.75
            The relevant atom site should be identified.
PLAT018_ALERT_1_C _diffrn_measured_fraction_theta_max .NE. *_full          ! Check
PLAT097_ALERT_2_C Large Reported Max.  (Positive) Residual Density       5.13 eA-3
PLAT202_ALERT_3_C Isotropic non-H Atoms in Anion/Solvent .........          4 Check
              O41     O48     O61     O94
PLAT220_ALERT_2_C NonSolvent Resd 1  O   Ueq(max) / Ueq(min) Range        5.5 Ratio
PLAT241_ALERT_2_C High   'MainMol' Ueq as Compared to Neighbors of        O11 Check
PLAT242_ALERT_2_C Low    'MainMol' Ueq as Compared to Neighbors of        Cu6 Check
```

---

### Alert level G

```
PLAT003_ALERT_2_G Number of Uiso or Uij Restrained non-H Atoms ...        167 Report
PLAT083_ALERT_2_G SHELXL Second Parameter in WGHT  Unusually Large      64.21 Why ?
PLAT168_ALERT_4_G The CIF-Embedded .res File Contains EXYZ Records          3 Report
PLAT171_ALERT_4_G The CIF-Embedded .res File Contains EADP Records          3 Report
PLAT186_ALERT_4_G The CIF-Embedded .res File Contains ISOR Records          1 Report
PLAT187_ALERT_4_G The CIF-Embedded .res File Contains RIGU Records          1 Report
PLAT300_ALERT_4_G Atom Site Occupancy of I1         Constrained at        0.8 Check
PLAT300_ALERT_4_G Atom Site Occupancy of I2         Constrained at       0.85 Check
PLAT300_ALERT_4_G Atom Site Occupancy of I3         Constrained at       0.85 Check
PLAT300_ALERT_4_G Atom Site Occupancy of O1M        Constrained at        0.2 Check
PLAT300_ALERT_4_G Atom Site Occupancy of O2M        Constrained at       0.15 Check
```

```
PLAT300_ALERT_4_G Atom Site Occupancy of O3M        Constrained at       0.15 Check
PLAT301_ALERT_3_G Main Residue  Disorder .............(Resd  1  )          2% Note
PLAT302_ALERT_4_G Anion/Solvent/Minor-Residue Disorder (Resd 29  )       100% Note
PLAT302_ALERT_4_G Anion/Solvent/Minor-Residue Disorder (Resd 30  )       100% Note
PLAT302_ALERT_4_G Anion/Solvent/Minor-Residue Disorder (Resd 31  )       100% Note
PLAT304_ALERT_4_G Non-Integer Number of Atoms in ..... (Resd 29  )       0.20 Check
PLAT304_ALERT_4_G Non-Integer Number of Atoms in ..... (Resd 30  )       0.15 Check
PLAT304_ALERT_4_G Non-Integer Number of Atoms in ..... (Resd 31  )       0.15 Check
PLAT311_ALERT_2_G Isolated Disordered Oxygen Atom (No H's ?) .....        O1M Check
PLAT311_ALERT_2_G Isolated Disordered Oxygen Atom (No H's ?) .....        O2M Check
PLAT311_ALERT_2_G Isolated Disordered Oxygen Atom (No H's ?) .....        O3M Check
PLAT606_ALERT_4_G VERY LARGE Solvent Accessible VOID(S) in Structure        ! Info
PLAT789_ALERT_4_G Atoms with Negative _atom_site_disorder_group  #          3 Check
PLAT790_ALERT_4_G Centre of Gravity not Within Unit Cell: Resd.  #          5 Note
              O
PLAT790_ALERT_4_G Centre of Gravity not Within Unit Cell: Resd.  #         25 Note
              O
PLAT794_ALERT_5_G Tentative Bond Valency for Nb1       (V)       .       4.87 Info
PLAT794_ALERT_5_G Tentative Bond Valency for Nb2       (V)       .       4.87 Info
PLAT794_ALERT_5_G Tentative Bond Valency for Nb4       (V)       .       4.95 Info
PLAT794_ALERT_5_G Tentative Bond Valency for Nb5       (V)       .       4.98 Info
PLAT794_ALERT_5_G Tentative Bond Valency for Nb6       (V)       .       4.91 Info
PLAT794_ALERT_5_G Tentative Bond Valency for Nb8       (V)       .       4.97 Info
PLAT794_ALERT_5_G Tentative Bond Valency for Nb9       (V)       .       4.84 Info
PLAT794_ALERT_5_G Tentative Bond Valency for Nb11      (V)       .       4.97 Info
PLAT794_ALERT_5_G Tentative Bond Valency for Nb12      (V)       .       4.91 Info
PLAT794_ALERT_5_G Tentative Bond Valency for Nb13      (V)       .       4.90 Info
PLAT794_ALERT_5_G Tentative Bond Valency for Nb14      (V)       .       4.95 Info
PLAT794_ALERT_5_G Tentative Bond Valency for Nb15      (V)       .       5.00 Info
PLAT794_ALERT_5_G Tentative Bond Valency for Nb16      (V)       .       4.90 Info
PLAT794_ALERT_5_G Tentative Bond Valency for Nb17      (V)       .       5.02 Info
PLAT794_ALERT_5_G Tentative Bond Valency for Nb18      (V)       .       4.96 Info
PLAT794_ALERT_5_G Tentative Bond Valency for Nb19      (V)       .       4.92 Info
PLAT794_ALERT_5_G Tentative Bond Valency for Nb20      (V)       .       4.96 Info
PLAT794_ALERT_5_G Tentative Bond Valency for Nb21      (V)       .       4.90 Info
PLAT794_ALERT_5_G Tentative Bond Valency for Nb22      (V)       .       4.98 Info
PLAT794_ALERT_5_G Tentative Bond Valency for Nb23      (V)       .       5.01 Info
PLAT794_ALERT_5_G Tentative Bond Valency for Nb24      (V)       .       4.91 Info
PLAT794_ALERT_5_G Tentative Bond Valency for Nb25      (V)       .       4.91 Info
PLAT794_ALERT_5_G Tentative Bond Valency for Nb26      (V)       .       4.97 Info
PLAT794_ALERT_5_G Tentative Bond Valency for Nb27      (V)       .       4.91 Info
PLAT794_ALERT_5_G Tentative Bond Valency for Nb28      (V)       .       4.92 Info
PLAT794_ALERT_5_G Tentative Bond Valency for Nb29      (V)       .       4.96 Info
PLAT794_ALERT_5_G Tentative Bond Valency for Nb31      (V)       .       4.95 Info
PLAT794_ALERT_5_G Tentative Bond Valency for Nb35      (V)       .       4.92 Info
PLAT794_ALERT_5_G Tentative Bond Valency for Cu6       (II)      .       2.07 Info
PLAT860_ALERT_3_G Number of Least-Squares Restraints ............        3700 Note
PLAT869_ALERT_4_G ALERTS Related to the Use of SQUEEZE Suppressed           ! Info
PLAT883_ALERT_1_G No Info/Value for _atom_sites_solution_primary .     Please Do !
PLAT941_ALERT_3_G Average HKL Measurement Multiplicity ...........        2.7 Low
PLAT965_ALERT_2_G The SHELXL WEIGHT Optimisation has not Converged     Please Check
```

---

0 **ALERT level A** = Most likely a serious problem - resolve or explain

29 **ALERT level B** = A potentially serious problem, consider carefully

7 **ALERT level C** = Check. Ensure it is not caused by an omission or oversight

60 **ALERT level G** = General information/check it is not something unexpected

4 ALERT type 1 CIF construction/syntax error, inconsistent or missing data

```
38 ALERT type 2 Indicator that the structure model may be wrong or deficient
 4 ALERT type 3 Indicator that the structure quality may be low
21 ALERT type 4 Improvement, methodology, query or suggestion
29 ALERT type 5 Informative message, check
```

---

It is advisable to attempt to resolve as many as possible of the alerts in all categories. Often the minor alerts point to easily fixed oversights, errors and omissions in your CIF or refinement strategy, so attention to these fine details can be worthwhile. In order to resolve some of the more serious problems it may be necessary to carry out additional measurements or structure refinements. However, the purpose of your study may justify the reported deviations and the more serious of these should normally be commented upon in the discussion or experimental section of a paper or in the "special_details" fields of the CIF. checkCIF was carefully designed to identify outliers and unusual parameters, but every test has its limitations and alerts that are not important in a particular case may appear. Conversely, the absence of alerts does not guarantee there are no aspects of the results needing attention. It is up to the individual to critically assess their own results and, if necessary, seek expert advice.

**Publication of your CIF in IUCr journals**

A basic structural check has been run on your CIF. These basic checks will be run on all CIFs submitted for publication in IUCr journals (*Acta Crystallographica*, *Journal of Applied Crystallography*, *Journal of Synchrotron Radiation*); however, if you intend to submit to *Acta Crystallographica Section C* or *E* or *IUCrData*, you should make sure that full publication checks are run on the final version of your CIF prior to submission.

**Publication of your CIF in other journals**

Please refer to the *Notes for Authors* of the relevant journal for any special instructions relating to CIF submission.

---

**PLATON version of 04/06/2020; check.def file version of 02/06/2020**

**Datablock 1** - ellipsoid plot

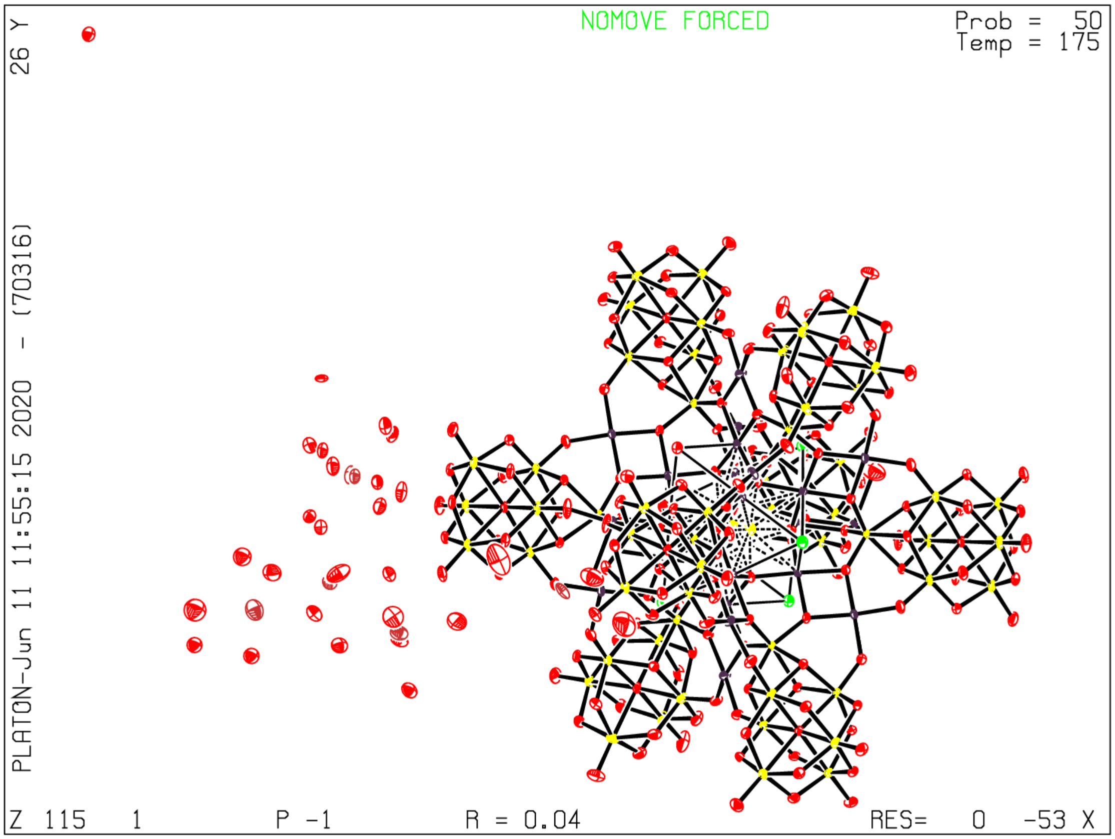

Supplement: Supplementary file 2 [file Data_Sheet_2.PDF]
