# Supplementary material for: A Series of Cube-Shaped Polyoxoniobates Encapsulating Octahedral Cu12XmOn Clusters With Hydrolytic Decomposition for Chemical Warfare Agents
Source: Front Chem. 2020 Dec 18;8:586009. doi: 10.3389/fchem.2020.586009 (PMC7775552; doi:10.3389/fchem.2020.586009)

checkCIF/PLATON report

Structure factors have been supplied for datablock(s) 1

THIS REPORT IS FOR GUIDANCE ONLY. IF USED AS PART OF A REVIEW PROCEDURE FOR PUBLICATION, IT SHOULD NOT REPLACE THE EXPERTISE OF AN EXPERIENCED CRYSTALLOGRAPHIC REFEREE.

No syntax errors found. CIF dictionary Interpreting this report

Datablock: 1

```
Bond precision:  Nb- O = 0.0049 A        Wavelength=0.71073

Cell:        a=21.7202(14)      b=26.8087(17)      c=32.885(2)
             alpha=92.1304(11)  beta=100.1557(11)  gamma=97.8603(11)
Temperature: 175 K
```

	Calculated	Reported
Volume	18635(2)	18634(2)
Space group	P -1	P -1
Hall group	-P 1	-P 1
Moiety formula	Br10 Cu48 Gd2 Nb112 O372, 22(O), 4(Na) [+ solvent]	?
Sum formula	Br10 Cu48 Gd2 Na4 Nb112 O394 [+ solvent]	Br5 Cu24 Gd Na2 Nb56 O197
Mr	20965.81	10482.70
Dx,g cm-3	1.868	1.868
Z	1	2
Mu (mm-1)	3.758	3.758
F000	9658.0	9658.0
F000’	9438.20	
h,k,lmax	25,31,39	25,31,38
Nref	65658	62801
Tmin,Tmax	0.316,0.400	0.193,0.259
Tmin’	0.292	

```
Correction method= # Reported T Limits: Tmin=0.193 Tmax=0.259
AbsCorr = MULTI-SCAN

Data completeness= 0.956          Theta(max)= 25.004

R(reflections)= 0.0455( 43943)    wR2(reflections)= 0.1461( 62801)

S = 1.024                   Npar= 2628
```

The following ALERTS were generated. Each ALERT has the format
test-name_ALERT_alert-type_alert-level.
Click on the hyperlinks for more details of the test.

Alert level B

```
PLAT306_ALERT_2_B Isolated Oxygen Atom (H-atoms Missing ?) .......        O1W Check
PLAT306_ALERT_2_B Isolated Oxygen Atom (H-atoms Missing ?) .......        O4W Check
PLAT306_ALERT_2_B Isolated Oxygen Atom (H-atoms Missing ?) .......        O5W Check
PLAT306_ALERT_2_B Isolated Oxygen Atom (H-atoms Missing ?) .......        O8W Check
PLAT306_ALERT_2_B Isolated Oxygen Atom (H-atoms Missing ?) .......       O11W Check
PLAT306_ALERT_2_B Isolated Oxygen Atom (H-atoms Missing ?) .......       O12W Check
PLAT306_ALERT_2_B Isolated Oxygen Atom (H-atoms Missing ?) .......       O13W Check
PLAT306_ALERT_2_B Isolated Oxygen Atom (H-atoms Missing ?) .......       O17W Check
PLAT306_ALERT_2_B Isolated Oxygen Atom (H-atoms Missing ?) .......       O24W Check
PLAT306_ALERT_2_B Isolated Oxygen Atom (H-atoms Missing ?) .......       O25W Check
PLAT910_ALERT_3_B Missing # of FCF Reflection(s) Below Theta(Min).         15 Note
PLAT934_ALERT_3_B Number of (Iobs-Icalc)/Sigma(W) > 10 Outliers ..          6 Check
PLAT975_ALERT_2_B Check Calcd Resid. Dens.  1.04A   From O8W             1.51 eA-3
PLAT990_ALERT_1_B Deprecated .res/.hkl Input Style SQUEEZE Job ...          ! Note
```

Alert level C

```
ABSTY02_ALERT_1_C  An _exptl_absorpt_correction_type has been given without
            a literature citation. This should be contained in the
            _exptl_absorpt_process_details field.
            Absorption correction given as multi-scan
PLAT018_ALERT_1_C _diffrn_measured_fraction_theta_max .NE. *_full           ! Check
PLAT094_ALERT_2_C Ratio of Maximum / Minimum Residual Density ....       2.64 Report
PLAT214_ALERT_2_C Atom O5W       (Anion/Solvent) ADP max/min Ratio        4.3 prolat
PLAT220_ALERT_2_C NonSolvent Resd 1  O   Ueq(max) / Ueq(min) Range        3.7 Ratio
PLAT241_ALERT_2_C High   'MainMol' Ueq as Compared to Neighbors of        O38 Check
PLAT241_ALERT_2_C High   'MainMol' Ueq as Compared to Neighbors of        O41 Check
PLAT911_ALERT_3_C Missing FCF Refl Between Thmin & STh/L=    0.595       2823 Report
PLAT976_ALERT_2_C Check Calcd Resid. Dens.  0.50A   From O8W            -0.88 eA-3
PLAT992_ALERT_5_C Repd & Actual _reflns_number_gt Values Differ by         25 Check
```

Alert level G

```
PLAT003_ALERT_2_G Number of Uiso or Uij Restrained non-H Atoms ...        282 Report
PLAT045_ALERT_1_G Calculated and Reported Z Differ by a Factor ...       0.50 Check
PLAT154_ALERT_1_G The s.u.'s on the Cell Angles are Equal ..(Note)     0.0011 Degree
PLAT168_ALERT_4_G The CIF-Embedded .res File Contains EXYZ Records          6 Report
PLAT171_ALERT_4_G The CIF-Embedded .res File Contains EADP Records          6 Report
PLAT186_ALERT_4_G The CIF-Embedded .res File Contains ISOR Records          1 Report
PLAT187_ALERT_4_G The CIF-Embedded .res File Contains RIGU Records          1 Report
PLAT300_ALERT_4_G Atom Site Occupancy of Br1        Constrained at       0.85 Check
PLAT300_ALERT_4_G Atom Site Occupancy of Br2        Constrained at       0.85 Check
PLAT300_ALERT_4_G Atom Site Occupancy of Br3        Constrained at       0.85 Check
PLAT300_ALERT_4_G Atom Site Occupancy of Br4        Constrained at        0.8 Check
PLAT300_ALERT_4_G Atom Site Occupancy of Br5        Constrained at       0.85 Check
PLAT300_ALERT_4_G Atom Site Occupancy of Br6        Constrained at        0.8 Check
PLAT300_ALERT_4_G Atom Site Occupancy of O1M        Constrained at       0.15 Check
PLAT300_ALERT_4_G Atom Site Occupancy of O2M        Constrained at       0.15 Check
PLAT300_ALERT_4_G Atom Site Occupancy of O3M        Constrained at       0.15 Check
PLAT300_ALERT_4_G Atom Site Occupancy of O4M        Constrained at        0.2 Check
PLAT300_ALERT_4_G Atom Site Occupancy of O5M        Constrained at       0.15 Check
PLAT300_ALERT_4_G Atom Site Occupancy of O6M        Constrained at        0.2 Check
PLAT301_ALERT_3_G Main Residue  Disorder ..............(Resd  1  )         2% Note
PLAT302_ALERT_4_G Anion/Solvent/Minor-Residue Disorder (Resd 12  )       100% Note
PLAT302_ALERT_4_G Anion/Solvent/Minor-Residue Disorder (Resd 13  )       100% Note
```

```
PLAT302_ALERT_4_G Anion/Solvent/Minor-Residue Disorder (Resd 14  )       100% Note
PLAT302_ALERT_4_G Anion/Solvent/Minor-Residue Disorder (Resd 15  )       100% Note
PLAT302_ALERT_4_G Anion/Solvent/Minor-Residue Disorder (Resd 16  )       100% Note
PLAT302_ALERT_4_G Anion/Solvent/Minor-Residue Disorder (Resd 17  )       100% Note
PLAT304_ALERT_4_G Non-Integer Number of Atoms in ..... (Resd 12  )       0.15 Check
PLAT304_ALERT_4_G Non-Integer Number of Atoms in ..... (Resd 13  )       0.15 Check
PLAT304_ALERT_4_G Non-Integer Number of Atoms in ..... (Resd 14  )       0.15 Check
PLAT304_ALERT_4_G Non-Integer Number of Atoms in ..... (Resd 15  )       0.20 Check
PLAT304_ALERT_4_G Non-Integer Number of Atoms in ..... (Resd 16  )       0.15 Check
PLAT304_ALERT_4_G Non-Integer Number of Atoms in ..... (Resd 17  )       0.20 Check
PLAT311_ALERT_2_G Isolated Disordered Oxygen Atom (No H’s ?) .....        O1M Check
PLAT311_ALERT_2_G Isolated Disordered Oxygen Atom (No H’s ?) .....        O2M Check
PLAT311_ALERT_2_G Isolated Disordered Oxygen Atom (No H’s ?) .....        O3M Check
PLAT311_ALERT_2_G Isolated Disordered Oxygen Atom (No H’s ?) .....        O4M Check
PLAT311_ALERT_2_G Isolated Disordered Oxygen Atom (No H’s ?) .....        O5M Check
PLAT311_ALERT_2_G Isolated Disordered Oxygen Atom (No H’s ?) .....        O6M Check
PLAT606_ALERT_4_G VERY LARGE Solvent Accessible VOID(S) in Structure        ! Info
PLAT790_ALERT_4_G Centre of Gravity not Within Unit Cell: Resd.  #         18 Note
              Na
PLAT794_ALERT_5_G Tentative Bond Valency for Nb1       (V)       .       5.02 Info
PLAT794_ALERT_5_G Tentative Bond Valency for Nb3       (V)       .       4.94 Info
PLAT794_ALERT_5_G Tentative Bond Valency for Nb4       (V)       .       4.88 Info
PLAT794_ALERT_5_G Tentative Bond Valency for Nb5       (V)       .       4.92 Info
PLAT794_ALERT_5_G Tentative Bond Valency for Nb6       (V)       .       4.91 Info
PLAT794_ALERT_5_G Tentative Bond Valency for Nb7       (V)       .       5.03 Info
PLAT794_ALERT_5_G Tentative Bond Valency for Nb8       (V)       .       4.84 Info
PLAT794_ALERT_5_G Tentative Bond Valency for Nb9       (V)       .       5.13 Info
PLAT794_ALERT_5_G Tentative Bond Valency for Nb10      (V)       .       4.96 Info
PLAT794_ALERT_5_G Tentative Bond Valency for Nb11      (V)       .       4.92 Info
PLAT794_ALERT_5_G Tentative Bond Valency for Nb12      (V)       .       5.02 Info
PLAT794_ALERT_5_G Tentative Bond Valency for Nb13      (V)       .       4.91 Info
PLAT794_ALERT_5_G Tentative Bond Valency for Nb14      (V)       .       4.97 Info
PLAT794_ALERT_5_G Tentative Bond Valency for Nb15      (V)       .       5.01 Info
PLAT794_ALERT_5_G Tentative Bond Valency for Nb16      (V)       .       4.89 Info
PLAT794_ALERT_5_G Tentative Bond Valency for Nb17      (V)       .       5.12 Info
PLAT794_ALERT_5_G Tentative Bond Valency for Nb18      (V)       .       4.82 Info
PLAT794_ALERT_5_G Tentative Bond Valency for Nb19      (V)       .       4.95 Info
PLAT794_ALERT_5_G Tentative Bond Valency for Nb20      (V)       .       4.99 Info
PLAT794_ALERT_5_G Tentative Bond Valency for Nb21      (V)       .       4.90 Info
PLAT794_ALERT_5_G Tentative Bond Valency for Nb22      (V)       .       4.85 Info
PLAT794_ALERT_5_G Tentative Bond Valency for Nb23      (V)       .       4.96 Info
PLAT794_ALERT_5_G Tentative Bond Valency for Nb24      (V)       .       4.88 Info
PLAT794_ALERT_5_G Tentative Bond Valency for Nb25      (V)       .       4.94 Info
PLAT794_ALERT_5_G Tentative Bond Valency for Nb26      (V)       .       4.99 Info
PLAT794_ALERT_5_G Tentative Bond Valency for Nb27      (V)       .       4.84 Info
PLAT794_ALERT_5_G Tentative Bond Valency for Nb28      (V)       .       4.95 Info
PLAT794_ALERT_5_G Tentative Bond Valency for Nb29      (V)       .       4.89 Info
PLAT794_ALERT_5_G Tentative Bond Valency for Nb30      (V)       .       4.99 Info
PLAT794_ALERT_5_G Tentative Bond Valency for Nb31      (V)       .       4.86 Info
PLAT794_ALERT_5_G Tentative Bond Valency for Nb33      (V)       .       5.04 Info
PLAT794_ALERT_5_G Tentative Bond Valency for Nb35      (V)       .       4.86 Info
PLAT794_ALERT_5_G Tentative Bond Valency for Nb36      (V)       .       4.85 Info
PLAT794_ALERT_5_G Tentative Bond Valency for Nb37      (V)       .       5.04 Info
PLAT794_ALERT_5_G Tentative Bond Valency for Nb38      (V)       .       4.95 Info
PLAT794_ALERT_5_G Tentative Bond Valency for Nb39      (V)       .       4.80 Info
PLAT794_ALERT_5_G Tentative Bond Valency for Nb40      (V)       .       4.87 Info
PLAT794_ALERT_5_G Tentative Bond Valency for Nb41      (V)       .       5.01 Info
PLAT794_ALERT_5_G Tentative Bond Valency for Nb42      (V)       .       5.05 Info
PLAT794_ALERT_5_G Tentative Bond Valency for Nb43      (V)       .       4.88 Info
PLAT794_ALERT_5_G Tentative Bond Valency for Nb44      (V)       .       4.93 Info
PLAT794_ALERT_5_G Tentative Bond Valency for Nb45      (V)       .       4.92 Info
PLAT794_ALERT_5_G Tentative Bond Valency for Nb46      (V)       .       4.86 Info
```

```
PLAT794_ALERT_5_G Tentative Bond Valency for Nb47      (V)       .       4.90 Info
PLAT794_ALERT_5_G Tentative Bond Valency for Nb48      (V)       .       4.93 Info
PLAT794_ALERT_5_G Tentative Bond Valency for Nb49      (V)       .       4.87 Info
PLAT794_ALERT_5_G Tentative Bond Valency for Nb50      (V)       .       4.82 Info
PLAT794_ALERT_5_G Tentative Bond Valency for Nb52      (V)       .       5.03 Info
PLAT794_ALERT_5_G Tentative Bond Valency for Nb53      (V)       .       5.15 Info
PLAT794_ALERT_5_G Tentative Bond Valency for Nb56      (V)       .       4.88 Info
PLAT794_ALERT_5_G Tentative Bond Valency for Nb57      (V)       .       4.93 Info
PLAT794_ALERT_5_G Tentative Bond Valency for Nb62      (V)       .       4.94 Info
PLAT794_ALERT_5_G Tentative Bond Valency for Nb63      (V)       .       4.91 Info
PLAT794_ALERT_5_G Tentative Bond Valency for Nb64      (V)       .       4.88 Info
PLAT794_ALERT_5_G Tentative Bond Valency for Nb65      (V)       .       4.87 Info
PLAT794_ALERT_5_G Tentative Bond Valency for Nb70      (V)       .       4.99 Info
PLAT860_ALERT_3_G Number of Least-Squares Restraints .............       8185 Note
PLAT869_ALERT_4_G ALERTS Related to the Use of SQUEEZE Suppressed           ! Info
PLAT883_ALERT_1_G No Info/Value for _atom_sites_solution_primary .     Please Do !
PLAT909_ALERT_3_G Percentage of I>2sig(I) Data at Theta(Max) Still        56% Note
PLAT933_ALERT_2_G Number of OMIT Records in Embedded .res File ...         10 Note
PLAT941_ALERT_3_G Average HKL Measurement Multiplicity ...........        1.8 Low
PLAT961_ALERT_5_G Dataset Contains no Negative Intensities .......     Please Check
PLAT965_ALERT_2_G The SHELXL WEIGHT Optimisation has not Converged     Please Check
```

```
   0 ALERT level A = Most likely a serious problem - resolve or explain
  14 ALERT level B = A potentially serious problem, consider carefully
  10 ALERT level C = Check. Ensure it is not caused by an omission or oversight
 104 ALERT level G = General information/check it is not something unexpected

   6 ALERT type 1 CIF construction/syntax error, inconsistent or missing data
  26 ALERT type 2 Indicator that the structure model may be wrong or deficient
   7 ALERT type 3 Indicator that the structure quality may be low
  31 ALERT type 4 Improvement, methodology, query or suggestion
  58 ALERT type 5 Informative message, check
```

It is advisable to attempt to resolve as many as possible of the alerts in all categories. Often the minor alerts point to easily fixed oversights, errors and omissions in your CIF or refinement strategy, so attention to these fine details can be worthwhile. In order to resolve some of the more serious problems it may be necessary to carry out additional measurements or structure refinements. However, the purpose of your study may justify the reported deviations and the more serious of these should normally be commented upon in the discussion or experimental section of a paper or in the "special_details" fields of the CIF. checkCIF was carefully designed to identify outliers and unusual parameters, but every test has its limitations and alerts that are not important in a particular case may appear. Conversely, the absence of alerts does not guarantee there are no aspects of the results needing attention. It is up to the individual to critically assess their own results and, if necessary, seek expert advice.

Publication of your CIF in IUCr journals

A basic structural check has been run on your CIF. These basic checks will be run on all CIFs submitted for publication in IUCr journals (*Acta Crystallographica*, *Journal of Applied Crystallography*, *Journal of Synchrotron Radiation*); however, if you intend to submit to *Acta Crystallographica Section C* or *E* or *IUCrData*, you should make sure that full publication checks are run on the final version of your CIF prior to submission.

Publication of your CIF in other journals

Please refer to the *Notes for Authors* of the relevant journal for any special instructions relating to CIF submission.

PLATON version of 04/06/2020; check.def file version of 02/06/2020

Datablock 1 - ellipsoid plot

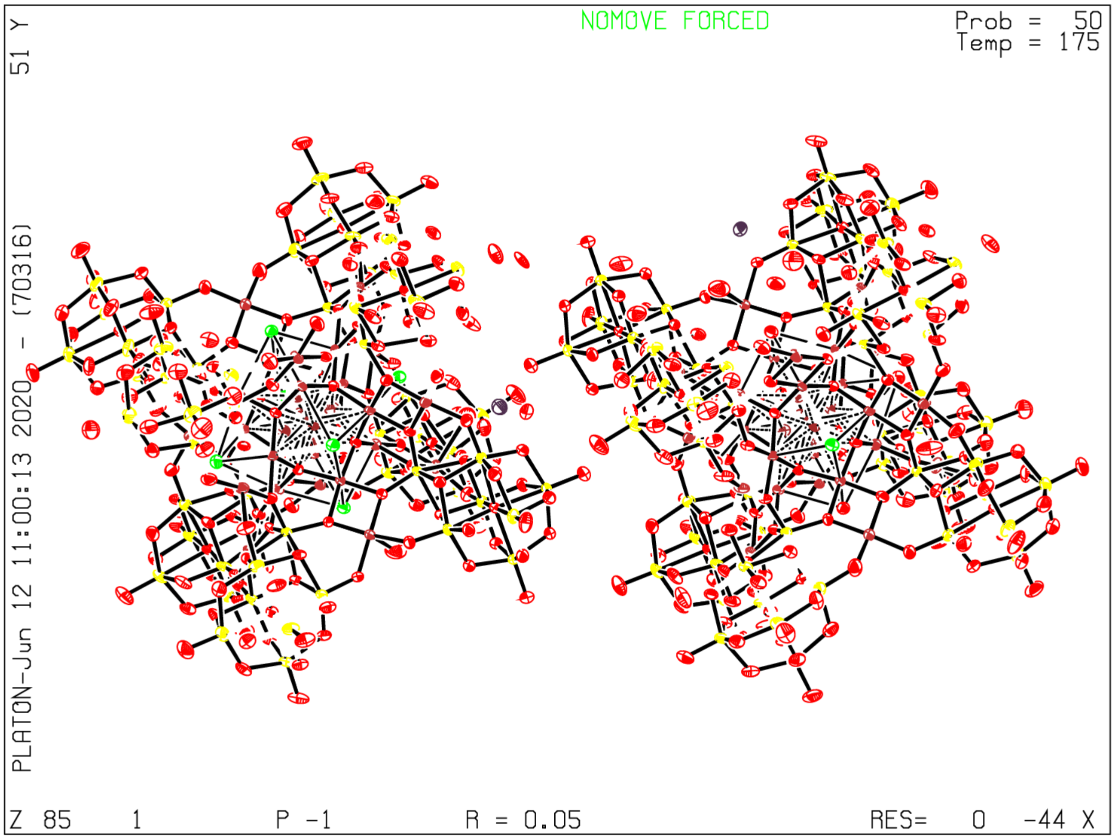

Supplement: Supplementary file 3 [file Data_Sheet_3.PDF]
