# Supplementary material for: A Series of Cube-Shaped Polyoxoniobates Encapsulating Octahedral Cu12XmOn Clusters With Hydrolytic Decomposition for Chemical Warfare Agents
Source: Front Chem. 2020 Dec 18;8:586009. doi: 10.3389/fchem.2020.586009 (PMC7775552; doi:10.3389/fchem.2020.586009)
Supplement: Supplementary file 4 [file Data_Sheet_4.pdf]

Supporting Information

A series of cube-shaped polyoxoniobates encapsulating octahedral $Cu_{12}X_mO_n$ clusters with hydrolytic decomposition for chemical warfare agents

Yan-Lan Wu, Rong-Tao Zhang, Yan-Qiong Sun,* Xin-Xiong Li and Shou-Tian Zheng*

State Key Laboratory of Photocatalysis on Energy and Environment, College of Chemistry, Fuzhou University, Fuzhou, Fujian 350108, China.

E-mail: sunyq@fzu.edu.cn; stzheng@fzu.edu.cn

Contents

1. Materials and Methods

2. Syntheses and Synthetic discussion

3. Single–crystal X–ray Crystallography

4. Supplementary Physical Characterization

5. Supplementary tables

(1) Table S1 Crystal Data and Structure Refinement for 1-3.

(2) Table S2 The bond lengths of central MO_8 cube-like unit in 1-3.

(3) Table S3 BVS calculation of protonated oxygen atoms of $\{Nb_7(OH)O_{21}\}^{8-}$ cluster in 1.

(4) Table S4 BVS calculation of protonated oxygen atoms of $\{Nb_7(OH)O_{21}\}^{8-}$ cluster in 2.

(5) Table S5 BVS calculation of protonated oxygen atoms of $\{Nb_7(OH)O_{21}\}^{8-}$ cluster in 3.

6. Additional Figures

1. Material and general methods.

All chemicals were commercially purchased and used without further purification. $K_7HNb_6O_{19}\cdot 13H_2O$ was prepared according to the literature and identified by IR spectrum.[S1] IR spectra were determined in the range 4000-400 cm^{-1} on a Nicolet IS50 Fourier transform infrared (FT/IR) spectrometer. ICP analyses were conducted on an Ultima2 spectrometer. XPS analyses were conducted on a Thermo Scientific Nexsa**.** ^{31}P NMR (202.46 MHz) spectroscopy conducted on an AVANCE III 400 MHz spectrometer, was used to detect the reagents and products from the catalytic reactions. Samples were placed in 5 mm O.D. NMR tubes and chemical shifts were referenced to H_3PO_4 (taken as 0 ppm at 25 ^oC). PXRD patterns were obtained by using an Ultima IV diffractometer with Cu-$K\alpha$ radiation (λ = 1.5418 Å) in the range 5-50°.

2. Syntheses and Synthetic discussion

(1) Synthesis of $Na_{12}\{H_{24}NbO_8Cu_{24}I_3O_3[Nb_7(OH)O_{21}]_8\}\cdot 34H_2O$ (1).

A mixture of $K_7HNb_6O_{19}\cdot 13H_2O$ (0.540 g, 0.394 mmol), Na_2CO_3 (0.067 g, 0.632 mmol), CuI (0.151 g, 0.793 mmol), $Gd(NO_3)_3\cdot 5H_2O$ (0.125 g, 0.288 mmol), $Na_3PO_4\cdot 12H_2O$ (0.160 g, 0.421 mmol) was mixed in 4 mL $Na_2B_4O_7/H_3BO_3$ buffer solution (0.05 M, pH = 9.11). After being stirred 1 hour, the resulting mixture was sealed in a Teflon-lined autoclave (23 mL) and heated at 100 ^oC for 5 days. After cooling to room temperature, brown crystals were obtained. Yield: 80 mg (16.7%, based on Nb). The pH values before and after reaction were ca. 9.11 and 9.5, respectively. Elemental analysis (based on dried sample) calcd (found) % for $H_{100}Na_{12}Nb_{57}Cu_{24}I_3O_{221}$: Na, 2.45 (2.48); Cu, 13.72 (13.67); I, 3.38 (3.42); Nb, 47.65 (47.50). IR (KBr, cm^{-1}): 3243(m), 1632(s), 1388(s), 1076(m), 1012(m), 864(s), 832(s), 769(w), 662(s), 530(w), 483(s).

(2) Synthesis of $Na_{12}(H_2O)_{22}\{H_{22}NbO_8Cu_{24}I_5O[Nb_7(OH)O_{21}]_8\}\cdot 34H_2O$ (2).

A mixture of $K_7HNb_6O_{19}\cdot 13H_2O$ (0.621 g, 0.453 mmol), $Li_2B_4O_7$ (0.067g, 0.396 mmol), Na_2CO_3 (0.181g, 1.707 mmol), CuI (0.082g, 0.431 mmol), tris(hydroxymethyl)methyl aminomethane (0.016g, 0.132 mmol) was mixd in 6 mL $Na_2CO_3/NaHCO_3$ buffer

solution (0.035 M, pH = 11.5), After stirred 1 hour, the resulting mixture was sealed in a Teflon-lined autoclave (23 mL) and heated at 140 °C for 5 days. After cooling to room temperature, brown crystals were obtained. Yield: 85 mg (15.3 %, based on Nb). The pH values before and after reaction were ca. 10.0 and 10.5, respectively. Elemental analysis (based on dried sample) calcd (found %) for $H_{142}Na_{12}Nb_{57}Cu_{24}I_5O_{241}$: Na, 2.32(2.35); Cu, 12.00 (13.01); I, 5.38 (5.40); Nb, 45.12 (45.15). IR (KBr, cm^{-1}): 3195(m), 1615(s), 1525(w), 1382(m), 1329(m), 858(s), 831(s), 767(w), 651(s), 529(w), 497(w), 465(s).

(3) Synthesis of $Na_2\{H_{34}GdO_8Cu_{24}Br_5O[Nb_7(OH)O_{21}]_8\} \cdot 12H_2O$ (3).

A mixture of $K_7HNb_6O_{19}\cdot 13H_2O$ (0.605 g, 0.441 mmol), $Na_2B_4O_7$ (0.071g, 0.353 mmol), Na_2CO_3 (0.089g, 0.839 mmol), CuBr (0.112g, 0.781 mmol), Gd_2O_3 (0.084g, 0.232 mmol) was mixed in 1 mL tris(hydroxymethyl)methyl aminomethane-HCl buffer solution (0.416 M, pH = 8.5) and 4 mL $Na_2CO_3/NaHCO_3$ buffer solution (0.035 M, pH = 10.5) and 2 mL $Na_2B_4O_7/H_3BO_3$ buffer solution (0.05 M, pH = 9.11), After being stirred 1 hour, the resulting mixture was sealed in a Teflon-lined autoclave (23 mL) and heated at 140 °C for 5 days. After cooling to room temperature, brown crystals were collected. Yield: 70 mg, (13.9 %, based on Nb). The pH values before and after reaction were ca. 10.0 and 9.0, respectively. Elemental analysis (based on dried sample) calcd (found %) for $H_{66}Na_2GdNb_{56}Cu_{24}Br_5O_{197}$: Na, 0.44 (0.43); Cu, 14.46 (14.46); Br, 3.79 (3.78); Gd, 1.49 (1.49); Nb, 49.32 (49.31). IR (KBr, cm^{-1}): 3258(m), 1638(s), 1416(s), 843(s), 769(w), 663(w), 530(w), 472(s).

(4). FT-IR spectrum of compounds 1, 2, 3.

The FT-IR spectrum of solid compound **1** were conducted on a Nicolet IS50 Fourier transform infrared spectrometer in the range 400-4000 cm^{-1} using KBr pellets (Fig. S18). In the low wavenumber range, four main typical vibration peaks appear at 483, 530, 662 and 769 cm^{-1}, which are assigned to the stretching vibrations of bridging Nb-O_b-Nb.[S2, S3] Four strong peaks at 832, 864, 1012 and 1076 cm^{-1}, are attributed to the vibration of the terminal Nb=O_t.[S4, S5] In the high wavenumber region, the strong and wide peak between 3000 cm^{-1} and 3500 cm^{-1} corresponds to the O-H stretching

vibration of lattice and coordination water molecules.[S6] These results are in good agreement with the result of the X-ray single-crystal structural analysis.

The FT-IR spectrum of solid compound **2** were conducted on a Nicolet IS50 Fourier transform infrared spectrometer in the range 400-4000 cm^{-1} using KBr pellets (Fig. S19). In the low wavenumber range, five main typical vibration peaks appear at 465, 497, 529, 651 and 767 cm^{-1}, which are assigned to the stretching vibrations of bridging Nb-O_b-Nb. Two strong peaks at 831 and 858 cm^{-1}, are attributed to the vibration of the terminal Nb=O_t. In the high wavenumber region, the strong and wide peak between 3000 cm^{-1} and 3500 cm^{-1} corresponds to the O-H stretching vibration of lattice and coordination water molecules. These results are in good agreement with the result of the X-ray single-crystal structural analysis.

The FT-IR spectrum of solid compound **3** were conducted on a Nicolet IS50 Fourier transform infrared spectrometer in the range 400-4000 cm^{-1} using KBr pellets (Fig. S20). In the low wavenumber range, four main typical vibration peaks appear at 472, 530, 663 and 769 cm^{-1}, which are assigned to the stretching vibrations of bridging Nb-O_b-Nb. The strong peak at 843 cm^{-1}, is attributed to the vibration of the terminal Nb=Ot. In the high wavenumber region, the strong and wide peak between 3000 cm^{-1} and 3500 cm^{-1} corresponds to the O-H stretching vibration of lattice and coordination water molecules. These results are in good agreement with the result of the X-ray single-crystal structural analysis.

(5). Synthetic Discussion

The buffer solution are is one of the important factors for the syntheses of compound **1**, **2** and **3**. Compound **1** was prepared in $Na_2B_4O_7/H_3BO_3$ buffer solution (0.05 M, pH = 9.11). Compound **2** was obtained in $Na_2CO_3/NaHCO_3$ buffer solution (0.035 M, pH = 11.5), while compound **3** was synthesized in three coexist buffer solutions, tris(hydroxymethyl)methyl aminomethane-HCl buffer solution (0.416 M, pH = 8.5) and 4 mL $Na_2CO_3/NaHCO_3$ buffer solution (0.035 M, pH = 10.5) and 2 mL $Na_2B_4O_7/H_3BO_3$ buffer solution (0.05 M, pH = 9.11).

3. Single−crystal X-ray Crystallography

Crystals were collected on a Bruker APEX Due CCD area diffractometer equipped with a fine focus, 2.0 kW sealed tube X-ray source (MoKα radiation, λ = 0.71073 Å) operating at 175(2) K. The empirical absorption correction was based on equivalent reflections. Structures were solved by direct methods followed by successive difference Fourier methods. Computations were performed using SHELXTL and final full-matrix refinements were against F^2.[S7] The contribution of disordered solvent molecules to the overall intensity data of structures were treated using the SQUEEZE method in PLATON. CSD1961427-1961429 contain the supplementary crystallographic data for compounds **1**, **2** and **3**, respectively.

4. Supplementary Physical Characterization

(1). Degradation of DMMP by compounds 1-3. In a typical reaction, 50 mg of compounds **1**, or **2**, or **3**, DMMP (2.5 μL) and D_2O (0.6 mL) were mixed with 1.0 mL of deionized water in a 2-mL scintillation vial. This mixture was vigorously stirred at room temperature. Every several hours, a 400 μL aliquot of the solvent was transferred into the NMR tube for ^{31}P NMR measurement; the aliquot was then transferred back to the vial for further reaction. After 264 hours, the solid was collected by centrifugation, washed extensively with mother liquid of compound **1-3** and dried, respectively. The resulting solid was then characterized by FT-IR.

(2). Degradation of DECP by compounds 1-3. In a typical reaction, 30 mg of compounds **1**, or **2** or **3** was suspended in 600 μL of DMF placed in a 5-mL O.D. NMR tube. Subsequently, 10 μL of DECP and 50 μL of H_2O were added. The mixture was vigorously shaken at room temperature. Every 5 minute, the contents were assessed by ^{31}P NMR spectroscopy to monitor the reaction. After the DECP has been completely hydrolyzed, the solid was collected by centrifugation, washed extensively with mother liquid of compound **1-3** and dried, respectively. The resulting solid was then characterized by FT-IR and XRD.

5. Supplementary tables

Table S1 Crystal Data and Structure Refinements for 1-3.

	**1**	**2**	**3**
Empirical formula	$Na_{12}\{H_{24}NbO_8Cu_{24}I_3O_3[Nb_7(OH)O_{21}]_8\}\cdot 34H_2O$	$Na_{12}(H_2O)_{22}\{H_{22}NbO_8Cu_{24}I_5O[Nb_7(OH)O_{21}]_8\}\cdot 34H_2O$	$Na_2\{H_{34}GdO_8Cu_{24}Br_5O[Nb_7(OH)O_{21}]_8\}\cdot 12H_2O$
M	11013.60	11587.42	10482.70
T/K	175(2)	175(2)	175(2)
Crystal system	Triclinic	Triclinic	Triclinic
Space group	$P\overline{1}$	$P\overline{1}$	$P\overline{1}$
a/Å	20.306(4)	20.284(3)	21.720(1)
b/Å	20.995(3)	21.050(2)	26.809(1)
c/Å	21.118(4)	21.098(3)	32.885(2)
α/°	80.025	79.997	92.130
β/°	88.583	88.551	100.155
γ/°	84.773	84.632	97.860
V/Å^3	8829(1)	8831.8(9)	18635(2)
Z	1	1	2
D_c/Mg m^{-3}	2.071	2.179	1.868
μ/mm^{-1}	3.530	3.712	3.758
F(000)	5092	5358	9658
Data/restraints/parameters	30673/3652/1463	31085/3700/1542	62801/8185/2628
$R_1(I > 2\sigma(I))$ [a]	0.0436	0.0394	0.0455
wR_2 (all data) [a]	0.1465	0.1136	0.1461
Goodness-of-fit on F^2	1.015	1.087	1.024

[a] $R_1 = \sum||F_0|-|F_C||/\sum|F_0|$; $wR_2 = \sum[w(F_0^2-F_C^2)^2]/\sum[w(F_0^2)^2]^{1/2}$

Table S2 The bond lengths of central MO_8 cube-like unit in 1-3.

	Nb(4A)-O(52)	1.965(6)

**1**	Nb(4A)-O(40)	1.994(4)
	Nb(3A)-O(52)	1.975(3)
	Nb(3A)-O(59)	1.987(3)
	Nb(3A)-O(40)	1.991(4)
	Nb(3A)-O(18)	2.018(3)
	Nb(5A)-O(52)	2.001(3)
	Nb(5A)-O(59)	2.002(4)
	Nb(37)- O(59)	2.386(4)
	Nb(37)- O(59#)	2.386(4)
	Nb(37)- O(52)	2.395(4)
	Nb(37)- O(52#)	2.395(4)
	Nb(37)- O(40)	2.414(4)
	Nb(37)- O(40#)	2.414(4)
	Nb(37)- O(18)	2.421(3)
	Nb(37)- O(18#)	2.421(4)
**2**	Nb(1A)-O(72)	1.956(6)
	Nb(1A)-O(15)	1.966(6)
	Nb(2A)-O(15)	1.945(4)
	Nb(2A)-O(39)	1.989(3)
	Nb(3A)-O(39)	1.934(5)
	Nb(3A)-O(72)	1.947(5)
	Nb(3A)-O(43)	1.954(5)
	Nb(3A)-O(15)	1.986(5)
	Nb(3)-O(72#)	2.344(4)
	Nb(3)-O(72)	2.344(4)
	Nb(3)-O(39#)	2.378(4)
	Nb(3)-O(39)	2.378(4)
	Nb(3)-O(15)	2.382(4)
	Nb(3)-O(15#)	2.382(4)
	Nb(3)-O(43#)	2.399(4)
	Nb(3)-O(43)	2.399(4)
**3**	Gd(1)-O(74)	2.377(4)
	Gd(1)-O(121)	2.380(4)
	Gd(1)-O(96)	2.391(4)
	Gd(1)-O(98)	2.398(4)
	Gd(1)-O(27)	2.400(4)
	Gd(1)-O(72)	2.400(4)
	Gd(1)-O(168)	2.410(4)
	Gd(1)-O(139)	2.416(4)

Table S3 BVS calculation of protonated oxygen atoms of $\{Nb_7(OH)O_{21}\}^{8-}$ cluster in 1.

Atom1	Atom 2	r_{ij}	r_0	B	S_{ij}	SUM

O40	Nb20	2.3099	1.921	0.319	0.295489	1.491264
	Nb25	2.3281	1.921	0.319	0.279103	
	Nb18	2.3342	1.921	0.319	0.273816	
	Nb38	2.4036	1.921	0.319	0.220281	
	Nb37	2.4089	1.921	0.319	0.216651	
	Nb22	2.4251	1.921	0.319	0.205923	
O46	Nb26	2.2751	1.921	0.319	0.329549	1.495377
	Nb28	2.2977	1.921	0.319	0.307009	
	Nb14	2.3621	1.921	0.319	0.250885	
	Nb29	2.3946	1.921	0.319	0.226584	
	Nb35	2.4284	1.921	0.319	0.216651	
	Nb33	2.4724	1.921	0.319	0.205923	
O68	Nb11	2.3096	1.921	0.319	0.295767	1.478616
	Nb10	2.3142	1.921	0.319	0.291533	
	Nb9	2.3304	1.921	0.319	0.277098	
	Nb12	2.4174	1.921	0.319	0.210954	
	Nb6	2.4274	1.921	0.319	0.204444	
	Nb5	2.4363	1.921	0.319	0.198819	
O79	Nb4	2.2947	1.921	0.319	0.30991	1.489238
	Nb16	2.2985	1.921	0.319	0.30624	
	Nb7	2.3297	1.921	0.319	0.277706	
	Nb17	2.4227	1.921	0.319	0.207479	
	Nb8	2.4366	1.921	0.319	0.198632	
	Nb15	2.4520	1.921	0.319	0.189271	

Table S4 BVS calculation of protonated oxygen atoms of $\{Nb_7(OH)O_{21}\}^{8-}$ cluster in 2.

Atom1	Atom 2	r_{ij}	r_0	B	S_{ij}	SUM
O42	Nb24	2.3020	1.921	0.319	0.302899	1.486928
	Nb19	2.3091	1.921	0.319	0.296231	
	Nb9	2.3399	1.921	0.319	0.268967	
	Nb21	2.4050	1.921	0.319	0.219316	
	Nb33	2.4291	1.921	0.319	0.219316	
	Nb18	2.4406	1.921	0.319	0.196157	
O83	Nb13	2.3097	1.921	0.319	0.295675	1.475898
	Nb6	2.3121	1.921	0.319	0.293459	
	Nb17	2.3144	1.921	0.319	0.29135	
	Nb14	2.4288	1.921	0.319	0.203549	
	Nb15	2.4318	1.921	0.319	0.201644	
	Nb16	2.4504	1.921	0.319	0.190222	
O89	Nb4	2.3099	1.921	0.319	0.295489	1.478245
	Nb7	2.3172	1.921	0.319	0.288804	
	Nb11	2.3128	1.921	0.319	0.292815	
	Nb8	2.4215	1.921	0.319	0.208261	

	Nb5	2.4422	1.921	0.319	0.195176	
	Nb12	2.4381	1.921	0.319	0.197700	
O94	Nb22	2.3104	1.921	0.319	0.295027	1.490836
	Nb26	2.3169	1.921	0.319	0.289076	
	Nb20	2.3203	1.921	0.319	0.286011	
	Nb31	2.4025	1.921	0.319	0.221042	
	Nb29	2.4240	1.921	0.319	0.206635	
	Nb27	2.4457	1.921	0.319	0.193046	

Table S5 BVS calculation of protonated oxygen atoms of $\{Nb_7(OH)O_{21}\}^{8-}$ cluster in 3.

Atom1	Atom 2	r_{ij}	r_0	B	S_{ij}	SUM
O119	Nb43	1.7982	1.921	0.319	1.469545	1.469545
O56	Nb40	2.3054	1.921	0.319	0.299687	1.489314
	Nb8	2.3173	1.921	0.319	0.288714	
	Nb13	2.3335	1.921	0.319	0.274418	
	Nb48	2.4118	1.921	0.319	0.214690	
	Nb43	2.4166	1.921	0.319	0.211484	
	Nb25	2.4339	1.921	0.319	0.200320	
O43	Nb36	1.8001	1.921	0.319	1.460818	1.460818
O18	Nb34	1.8235	1.921	0.319	1.357497	1.490836
O17	Nb15	1.7987	1.921	0.319	1.467244	1.467244
O6	Nb22	1.8208	1.921	0.319	1.369036	1.369036
O188	Nb37	1.7930	1.921	0.319	1.493696	1.493696
O189	Nb41	1.7945	1.921	0.319	1.486689	1.486689

6. Additional Figures

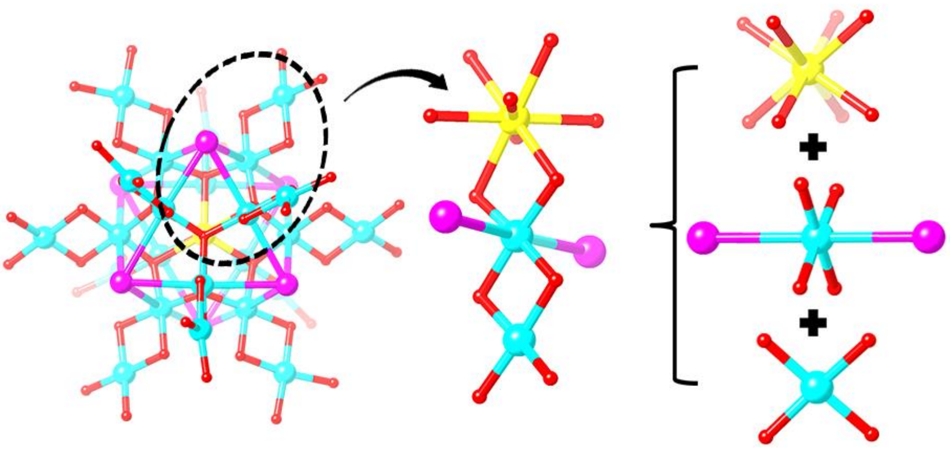

Fig. S1 Three types coordination modes of Cu atoms in compound **1**. Color code: M, yellow; Cu, cyan; I/O, purple; O, red.

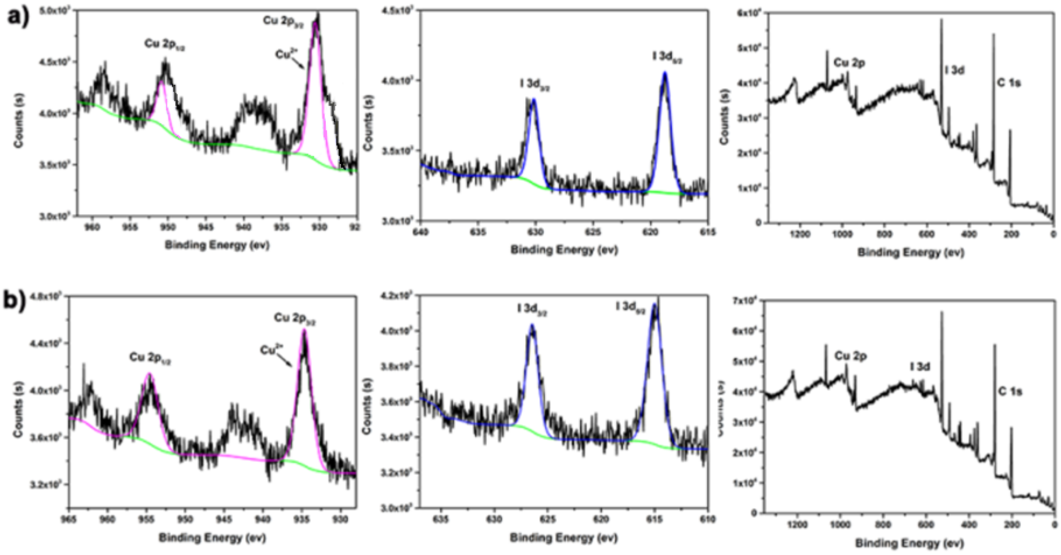

Fig. S2 XPS spectra of compounds **1** (**a**) and **2** (**b**).

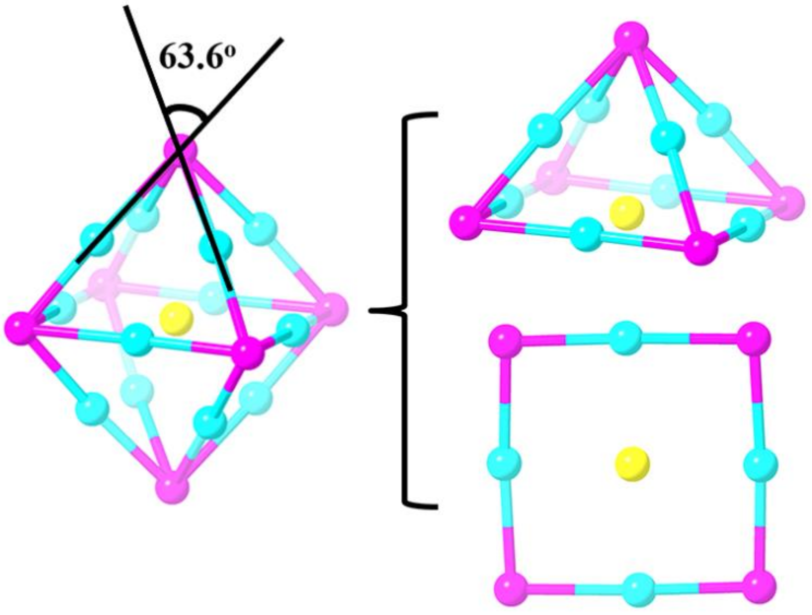

Fig. S3 View of the structure of the $\{Cu_{12}X_mO_n\}$ octahedron. Color code: M, yellow; Cu, cyan; I/O, purple.

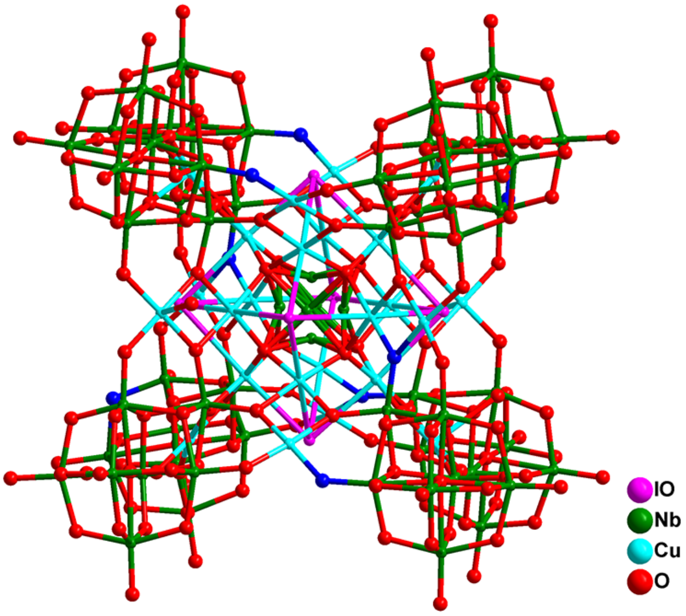

Fig. S4 The structure of $\{(NbO_8Cu_{24}I_3O_3)[Nb_7(OH)O_{21}]_8\}$ cluster in **1**, showing the protonated oxygen (blue). Color code: Cu, cyan; Nb, dark green; I/O, purple; O, red.

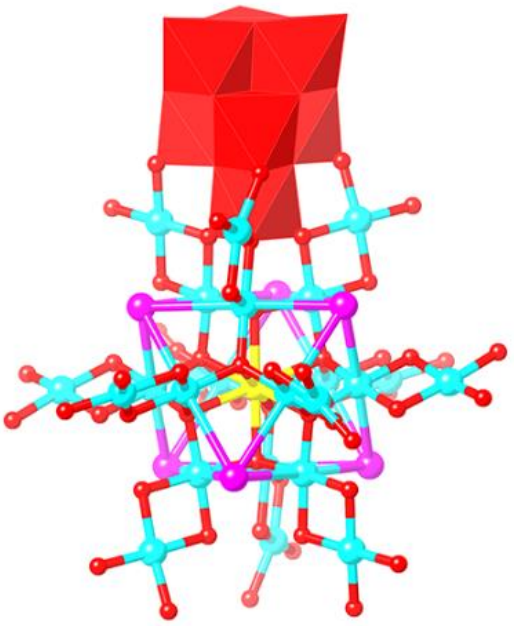

Fig. S5 Linkages between Cu atoms and $\{(Nb_7(OH)O_{21})_8\}^{8-}\}$ cluster units in compound **1**. Color code: M, yellow; Cu, cyan; I/O, purple; O, red.

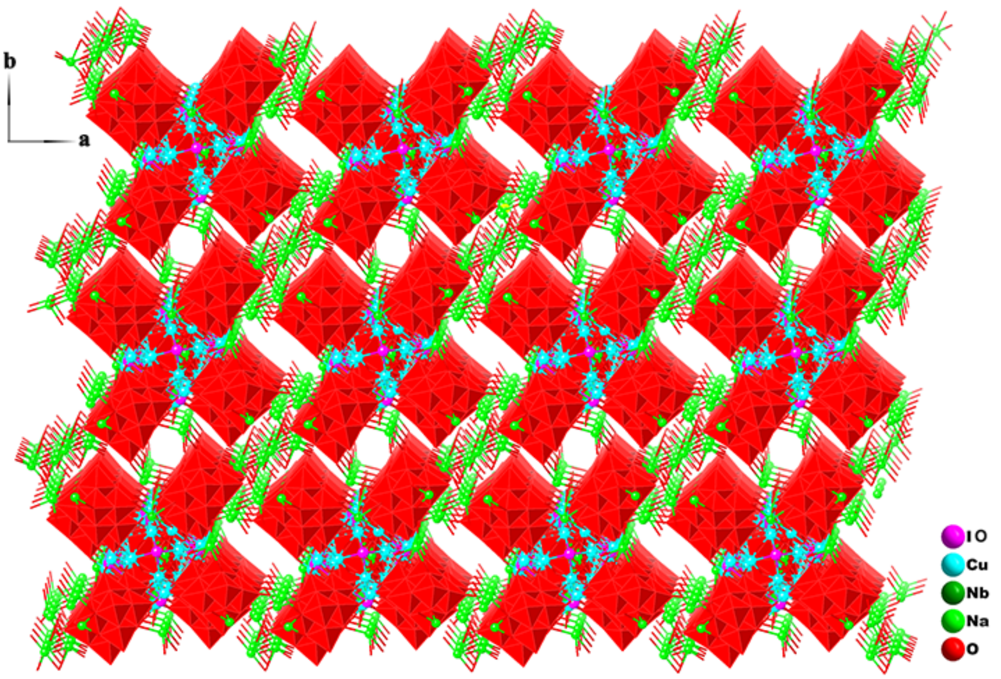

Fig. S6 The 3D stacking structure in compound **1**. Color code: Cu, cyan; Na, green; I/O, purple; O, red; NbO_6, red octahedron.

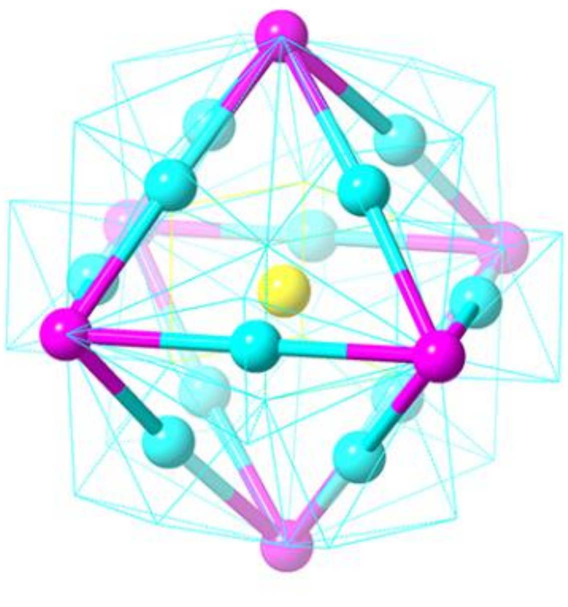

Fig. S7 The structure of cluster $\{NbO_8Cu_{12}I_5O\}$ in compound **2**, Color code: M, yellow; Cu, cyan; Na, green; I/O, purple; O, red.

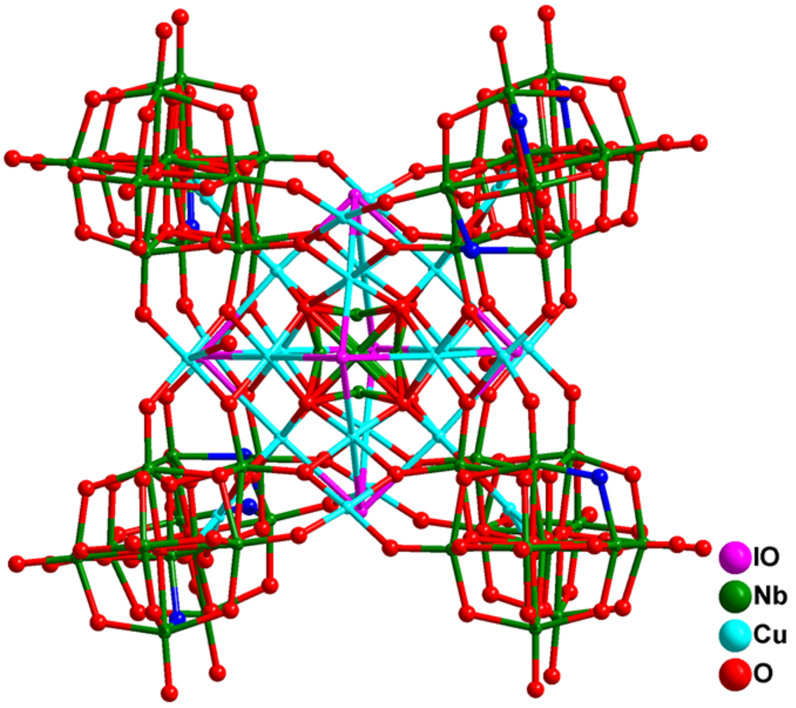

Fig. S8 The structure of {$(NbO_8Cu_{24}I_5O)[Nb_7(OH)O_{21}]_8$} cluster in **2**, showing the protonated oxygen (blue). Color code: Cu, cyan; Na, green; Nb, dark green; I/O, purple; O, red.

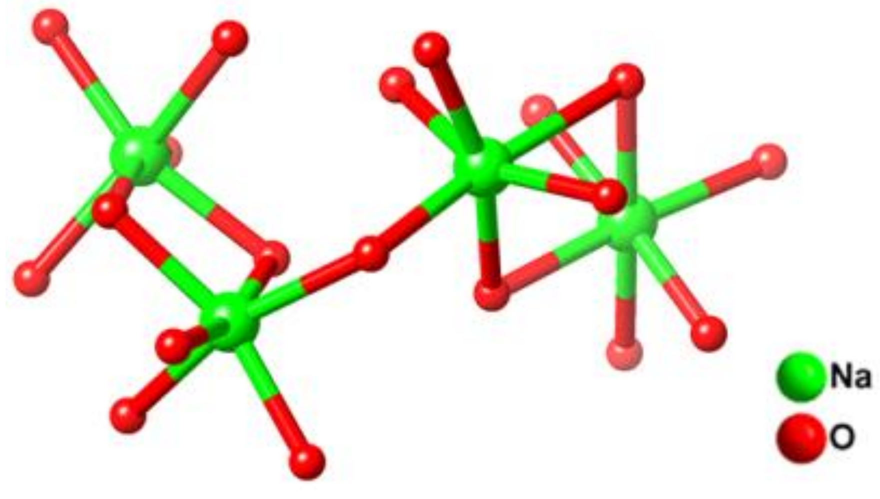

Fig. S9 The structure of {$Na_4(H_2O)_{19}$} cluster in compound **2**.

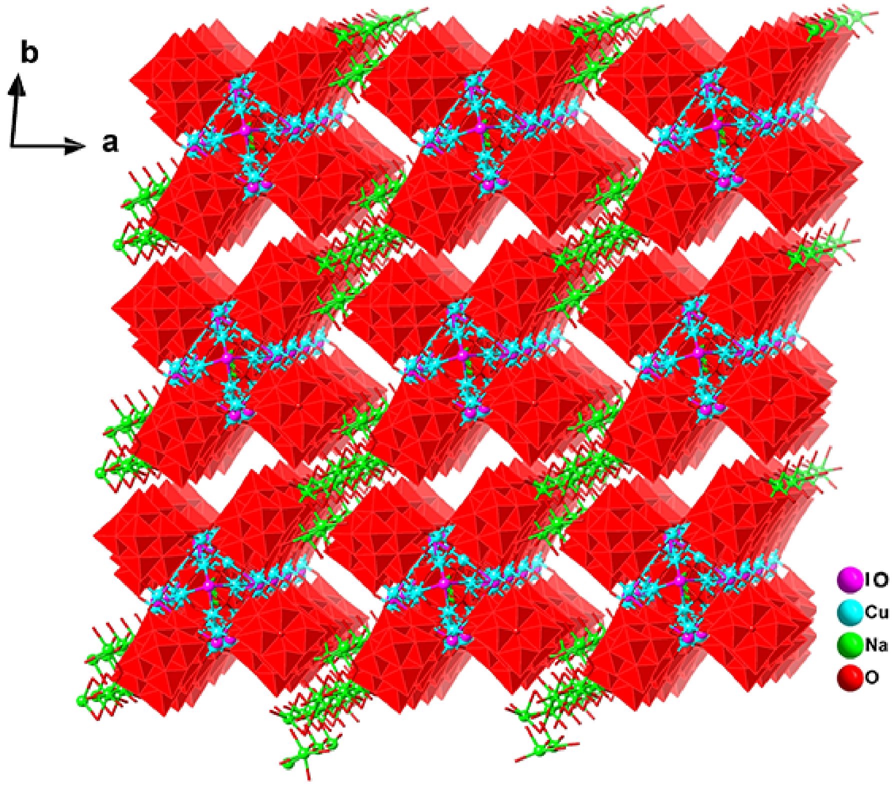

Fig. S10 The 3D stacking structure in compound **2**. Color code: Cu, cyan; Na, green; I/O, purple; O, red; NbO_6, red octahedron.

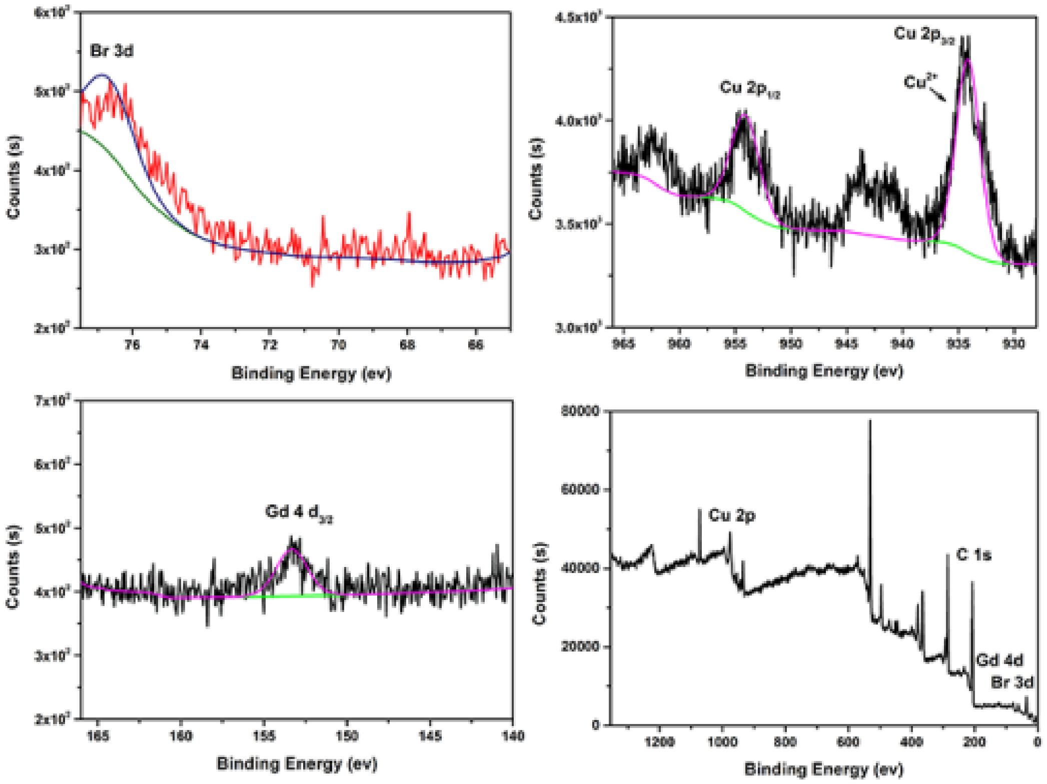

Fig. S11 XPS spectra of compound **3**.

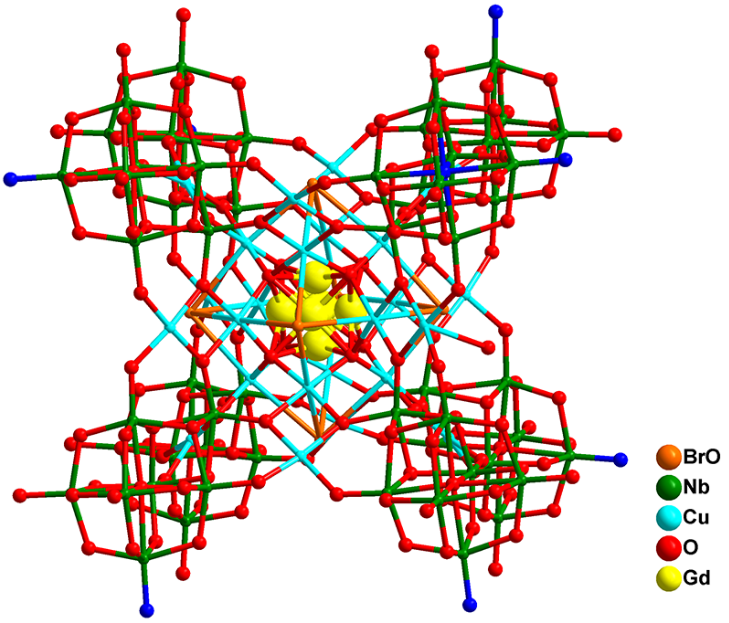

Fig. S12 The structure of {($GdO_8Cu_{24}Br_5O$)[$Nb_7(OH)O_{21}$]$_8$} cluster in **3**, showing the protonated oxygen (blue). Color code: Cu, cyan; Na, green; Nb, dark green; Gd, yellow; Br/O, deep yellow; O, red.

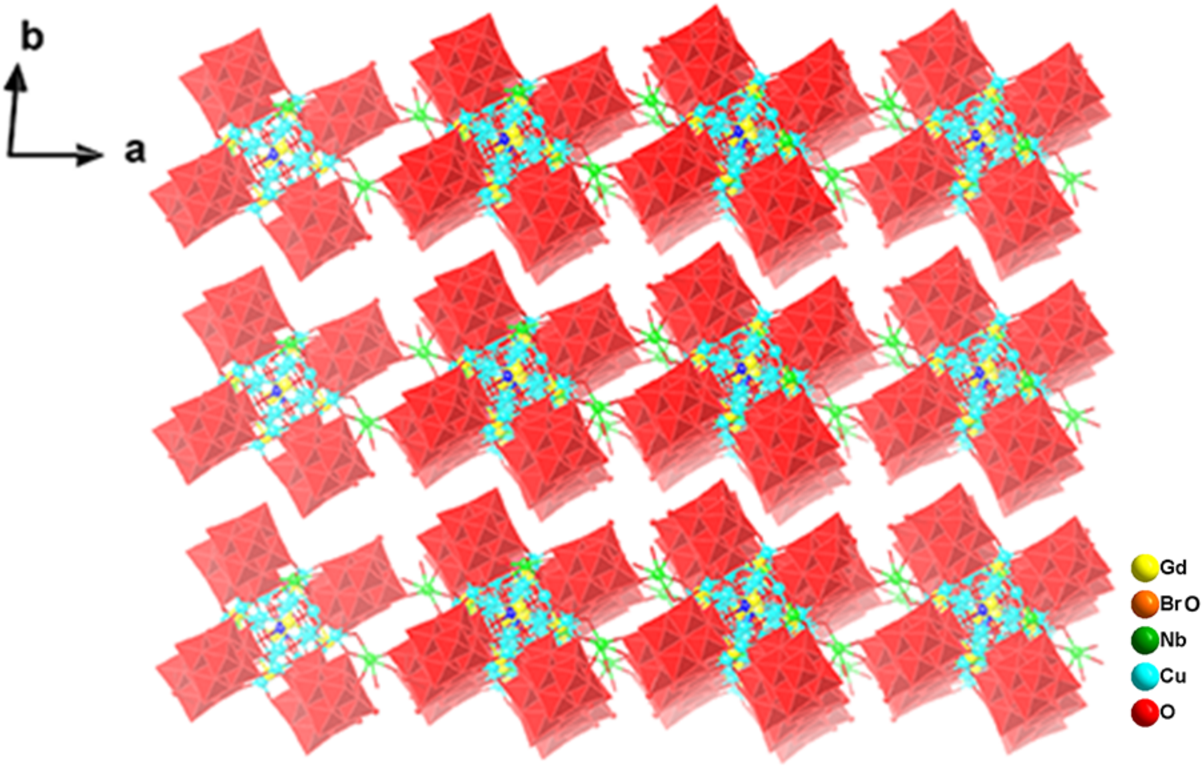

Fig. S13 The 3D stacking structure in compound **3**. Color code: Gd, yellow; Cu, cyan; Na, green; Br/O, deep yellow; O, red; NbO_6, red octahedron.

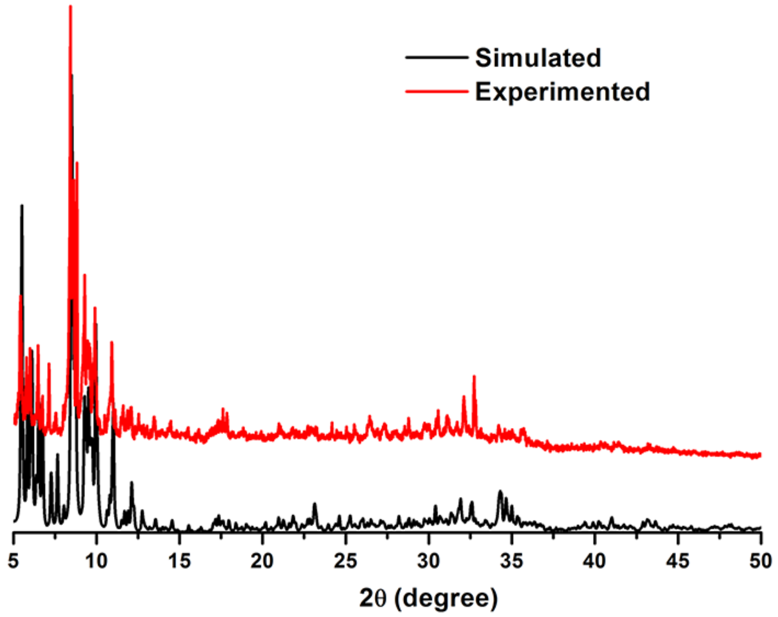

Fig. S14 The simulated and experimental PXRD patterns of of **1**.

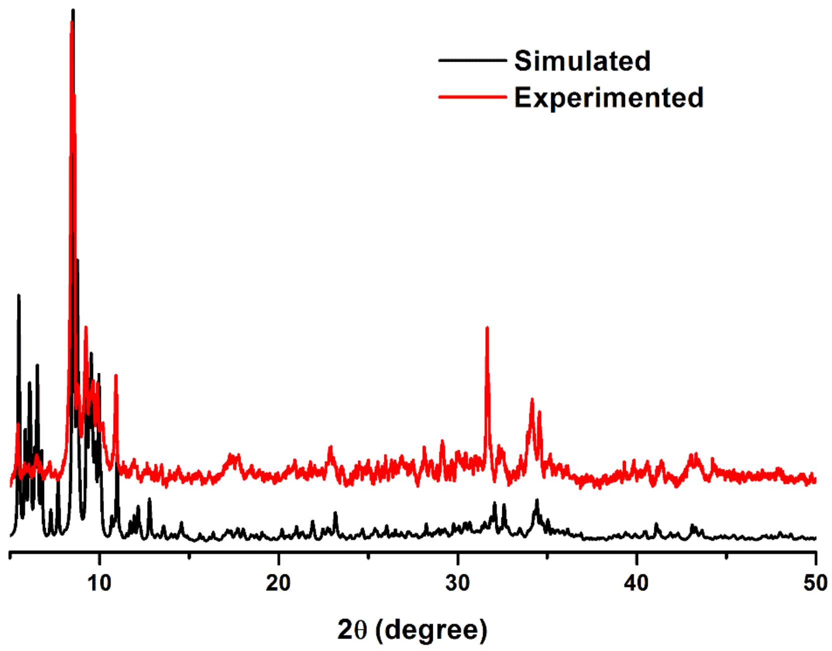

Fig. S15 The simulated and experimental PXRD patterns of of **2**.

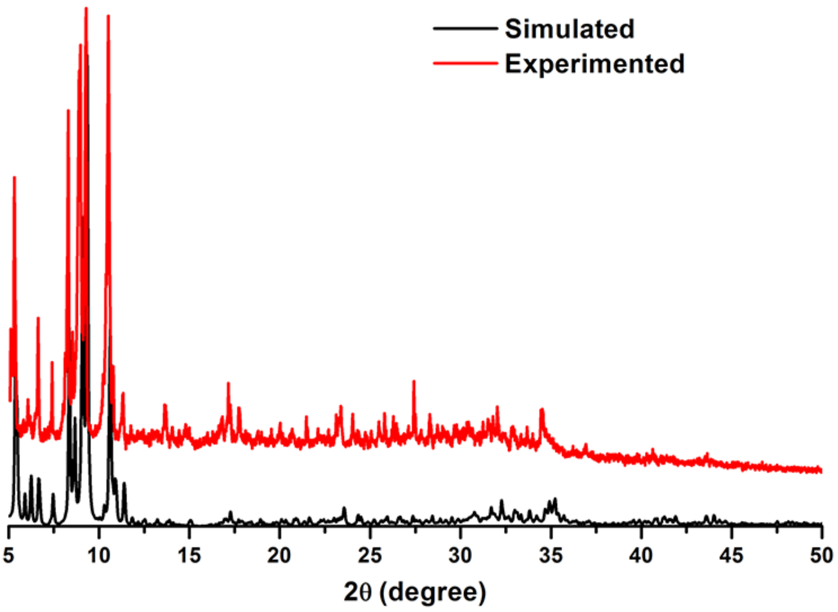

Fig. S16 The simulated and experimental PXRD patterns of of **3**.

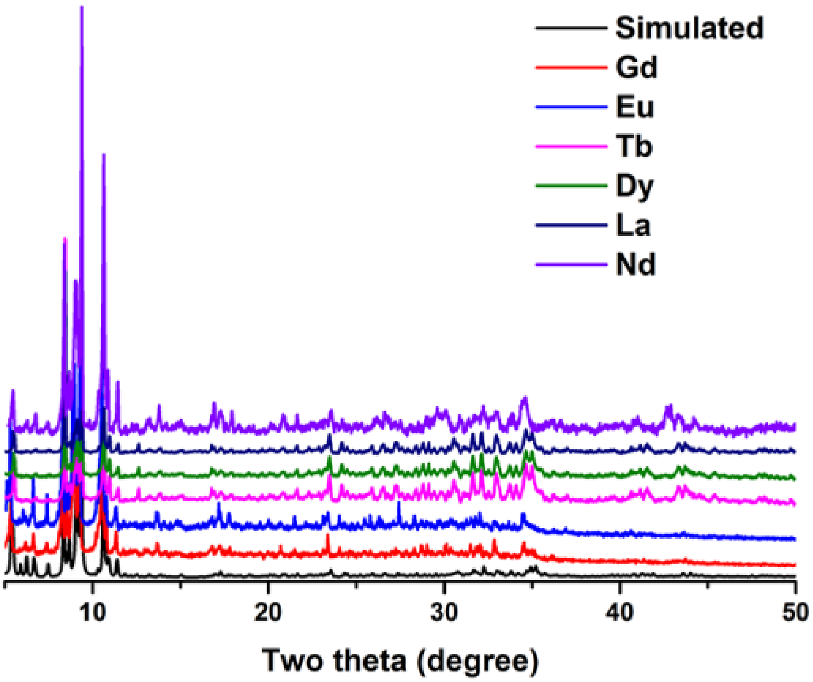

Fig. S17 The simulated and experimental PXRD patterns of $Na_2\{H_{34}LnO_8Cu_{24}Br_5O\}[Nb_7(OH)O_{21}]_8\cdot 12H_2O$ (**3,** Ln = Gd, Eu, Tb, Dy).

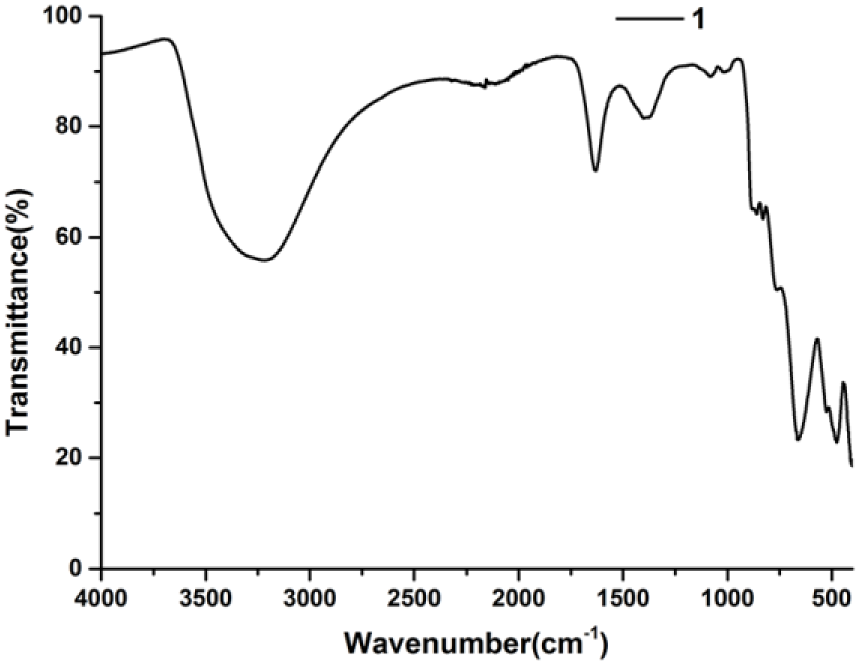

Fig. S18 IR Spectrum of **1**.

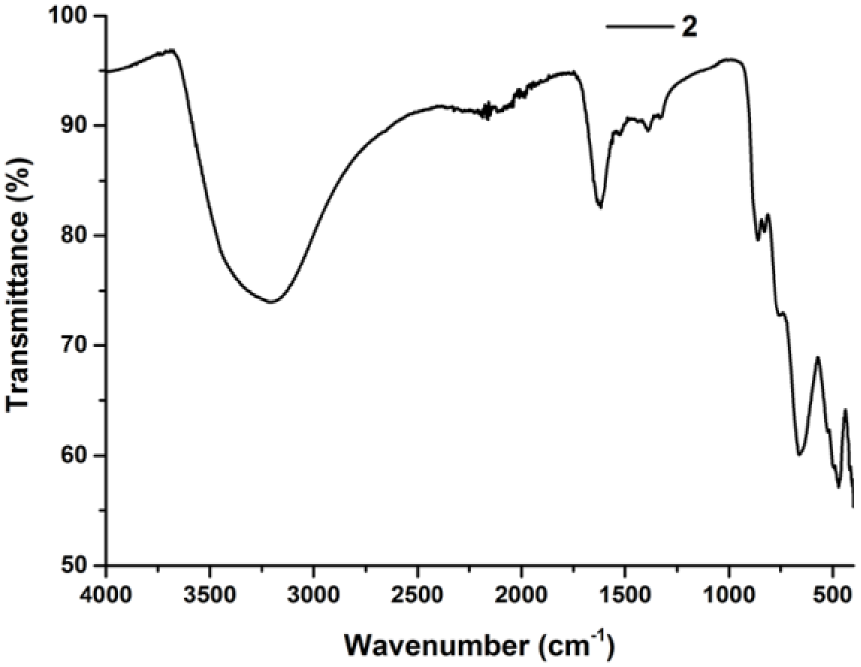

Fig. S19 IR Spectrum of **2**.

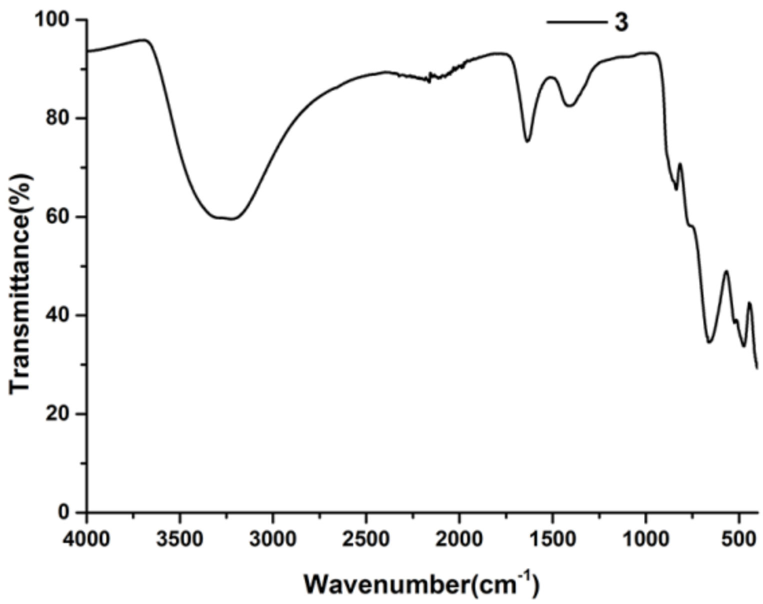

Fig. S20 IR Spectrum of **3**.

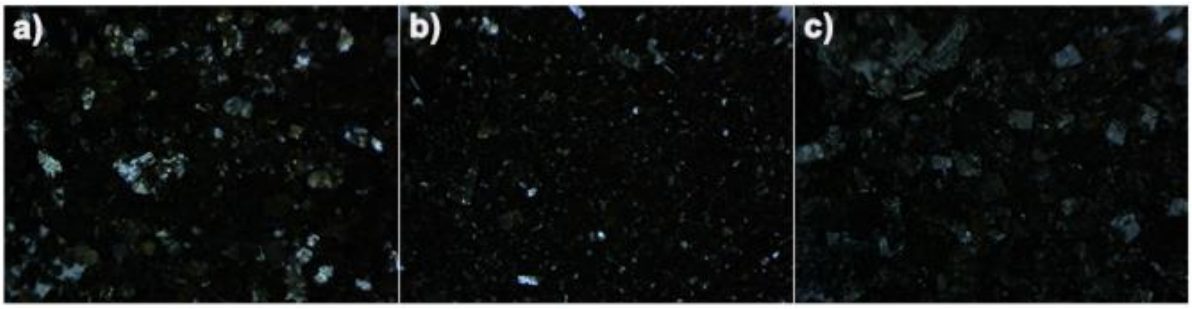

Fig. S21 Optical microscope photograph of compound **1 (a)**, **2 (b)** and **3 (c)**.

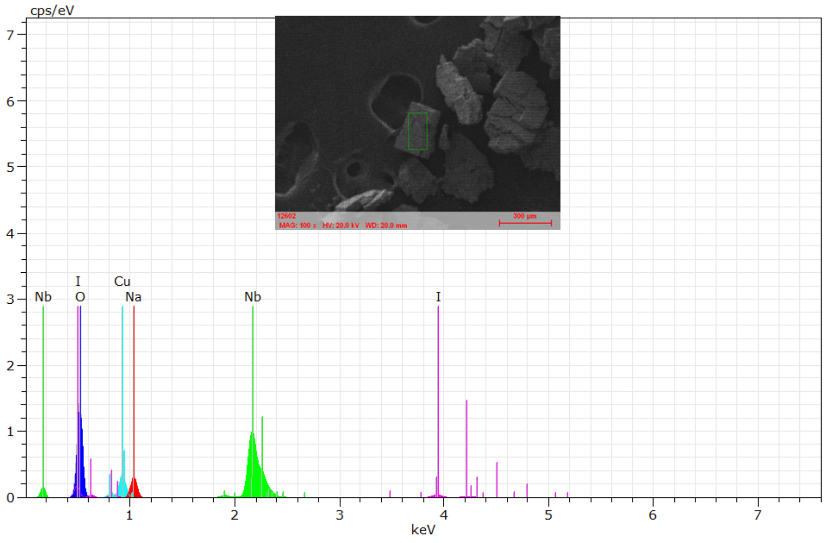

Fig. S22 EDS of **1**.

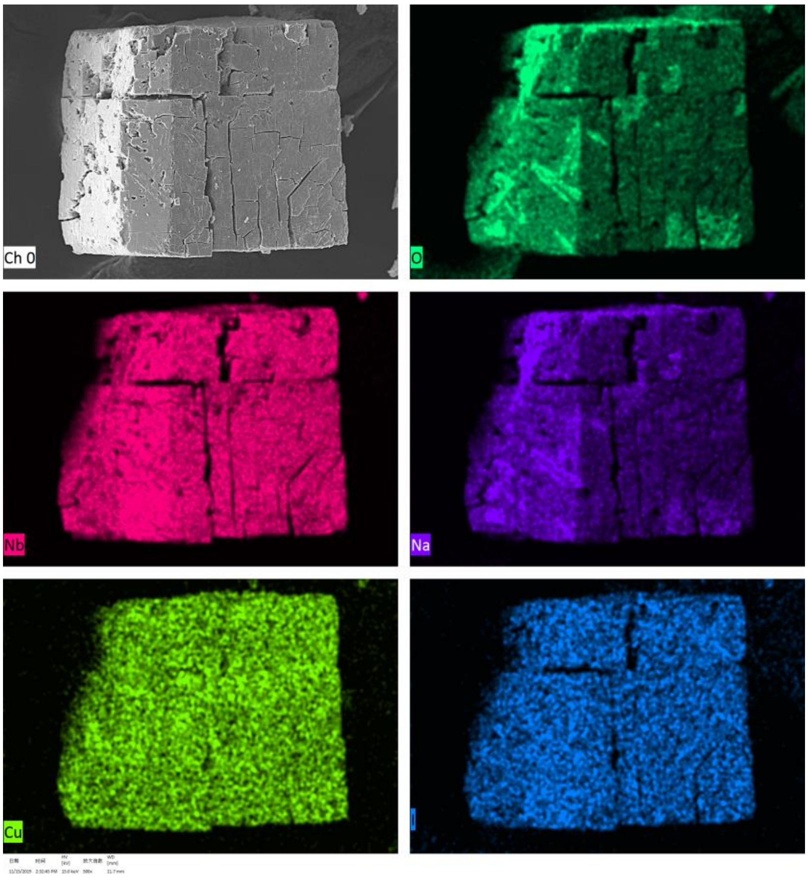

Fig. S23 EDS-mapping of **1**.

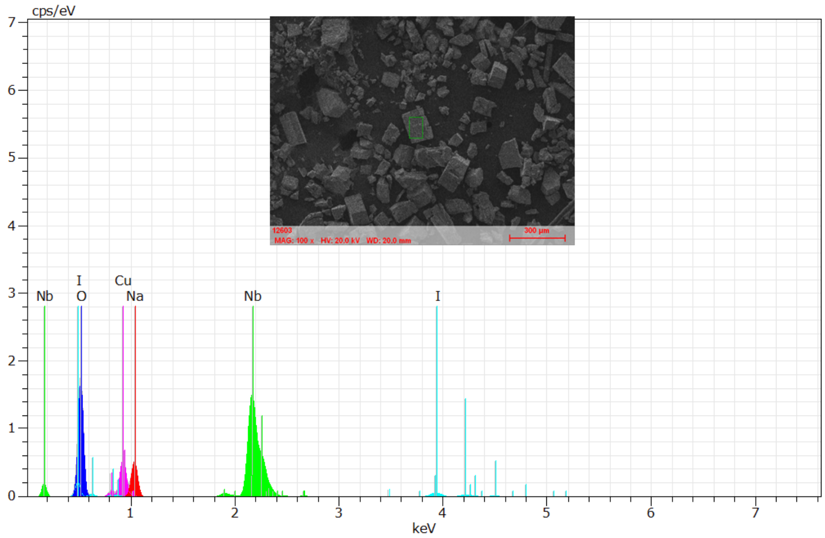

Fig. S24 EDS pattern of **2**.

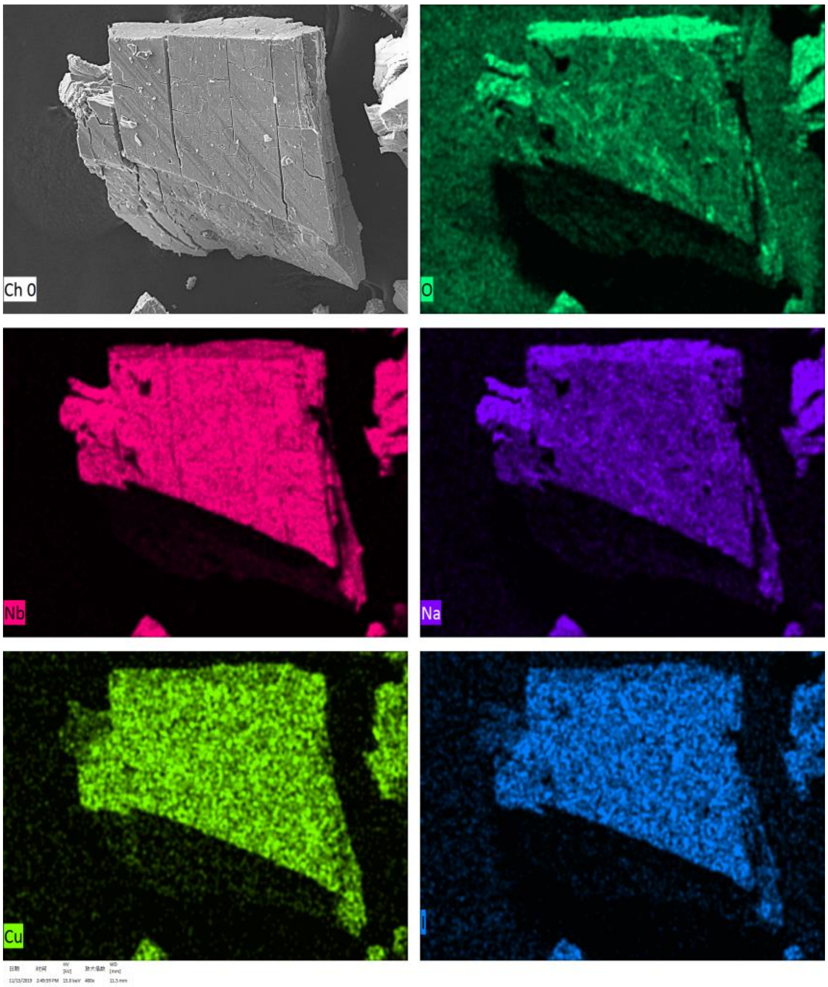

Fig. S25 EDS-mapping of **2**.

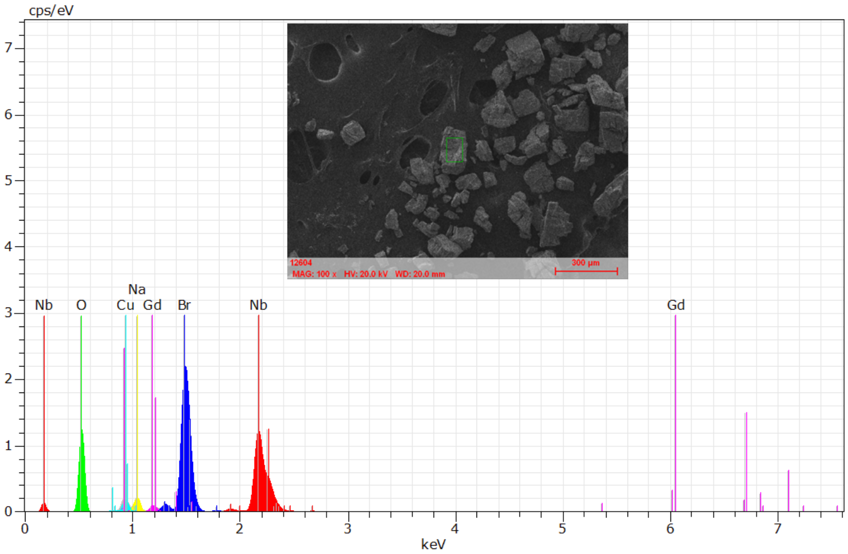

Fig. S26 EDS pattern of **3**.

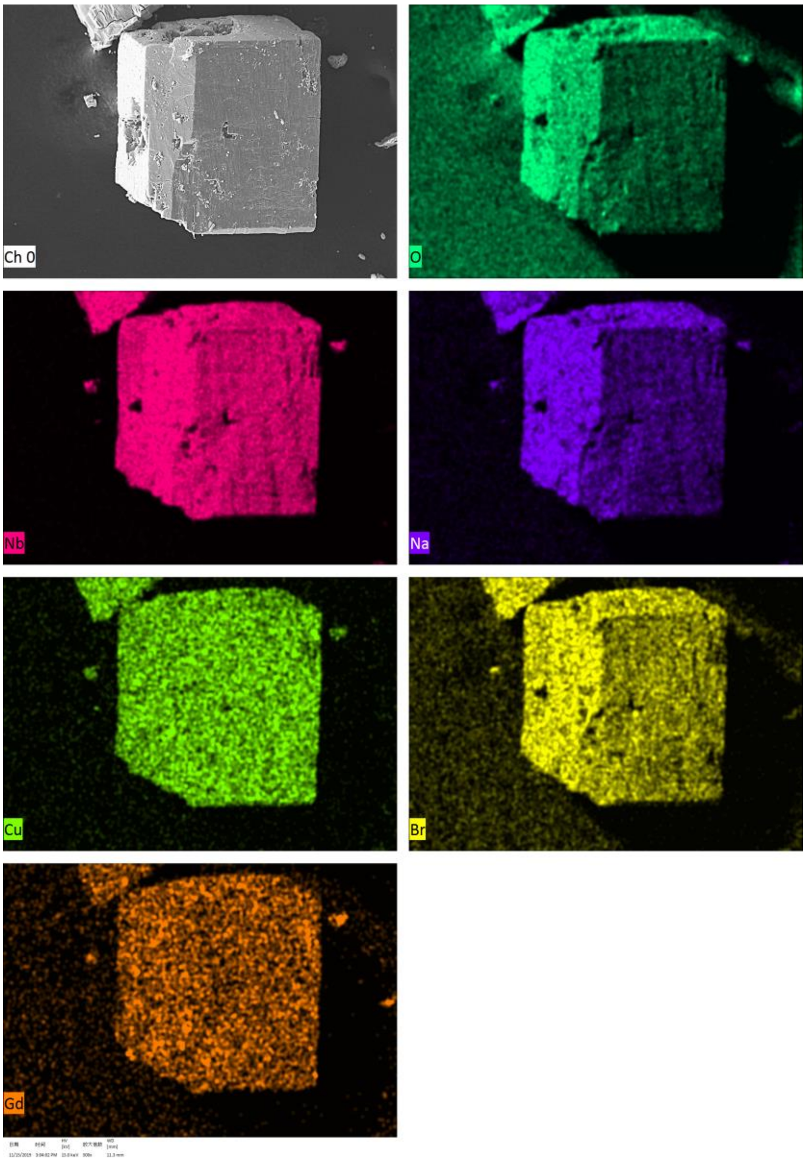

Fig. S27 EDS-mapping of **3**.

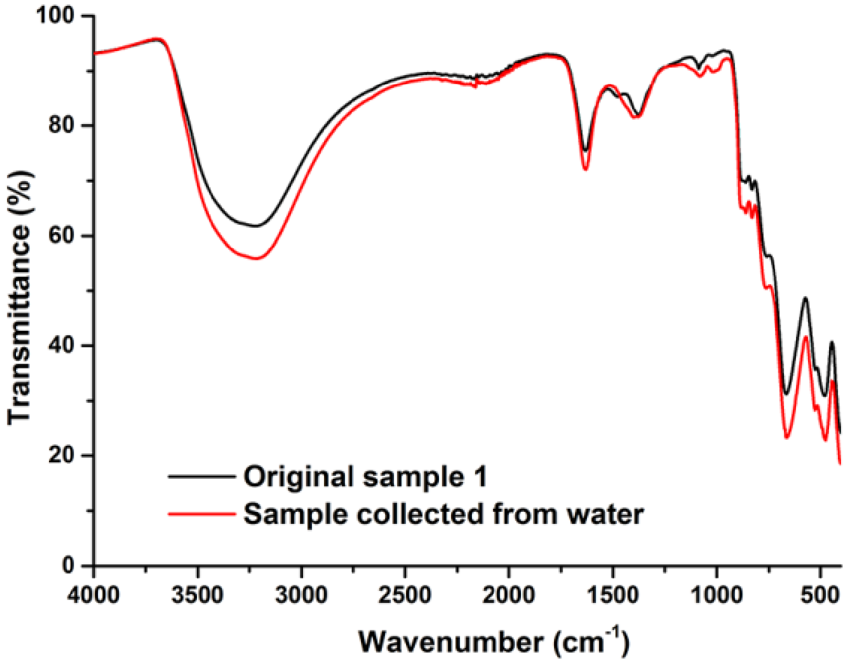

Fig. S28 The IR spectra of **1** before and after immersion in water.

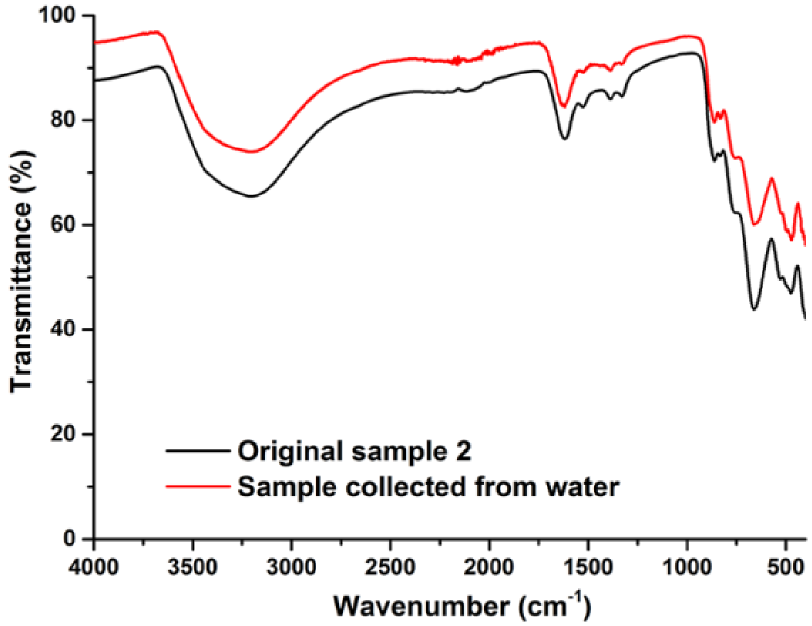

Fig. S29 The IR spectra of **2** before and after immersion in water.

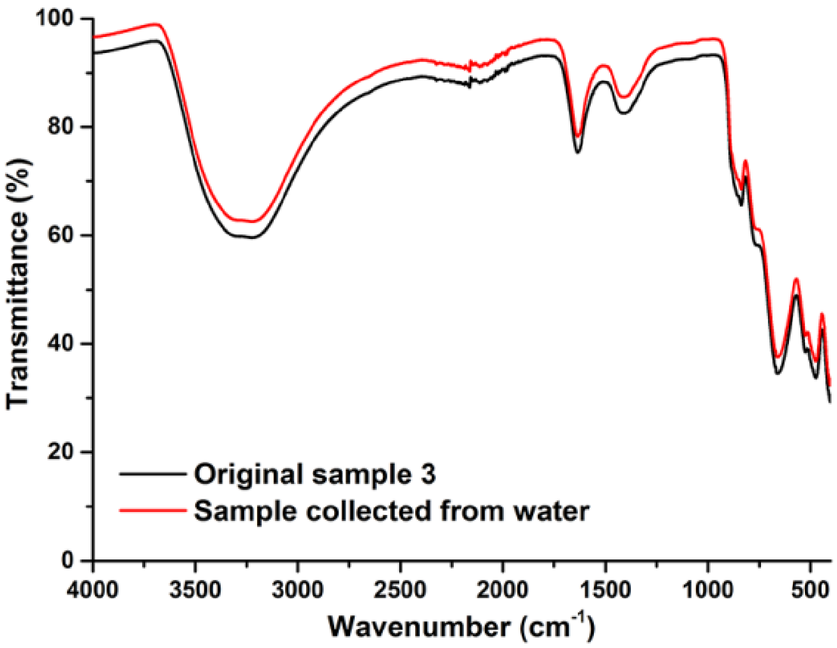

Fig. S30 The IR spectra of **3** before and after immersion in water.

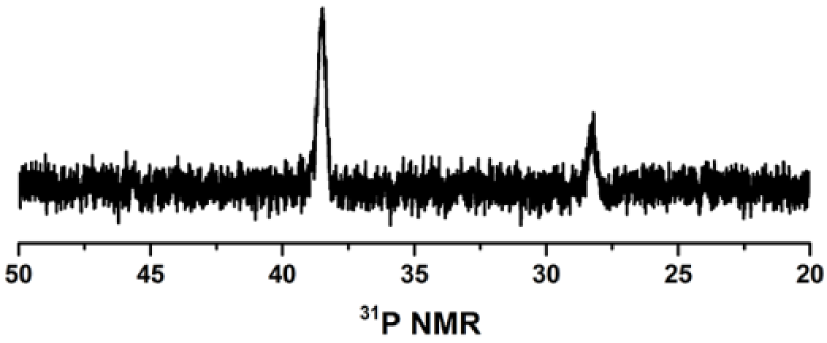

Fig. S31 ^{31}P NMR spectrum of DMMP decomposition by compound **1** after 24h in 1 mL H_2O and 0.6 mL D_2O.

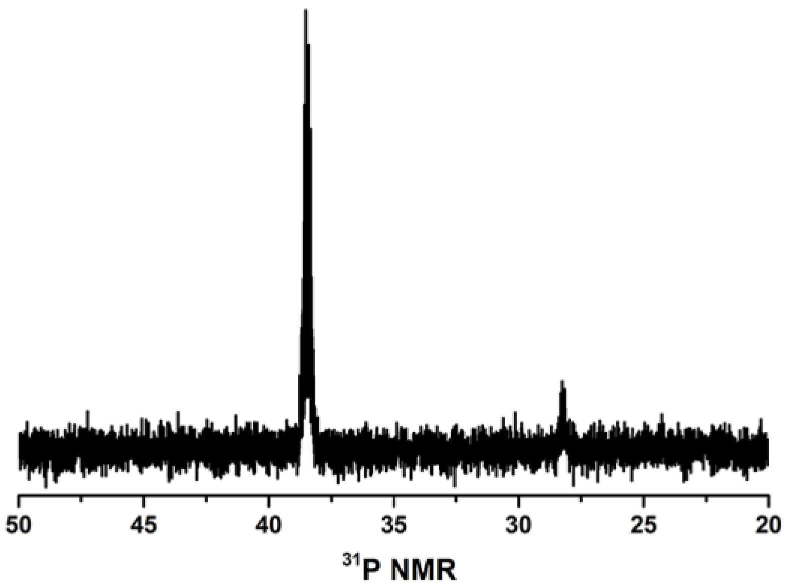

Fig. S32 ^{31}P NMR spectrum of DMMP decomposition by compound **2** after 24h in 1 mL H_2O and 0.6 mL D_2O.

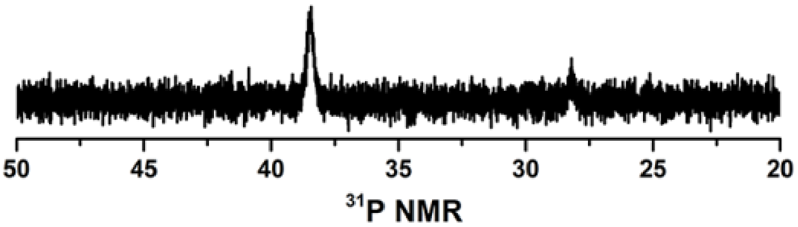

Fig. S33 ^{31}P NMR spectrum of DMMP decomposition by compound **3** after 24h in 1 mL H_2O and 0.6 mL D_2O.

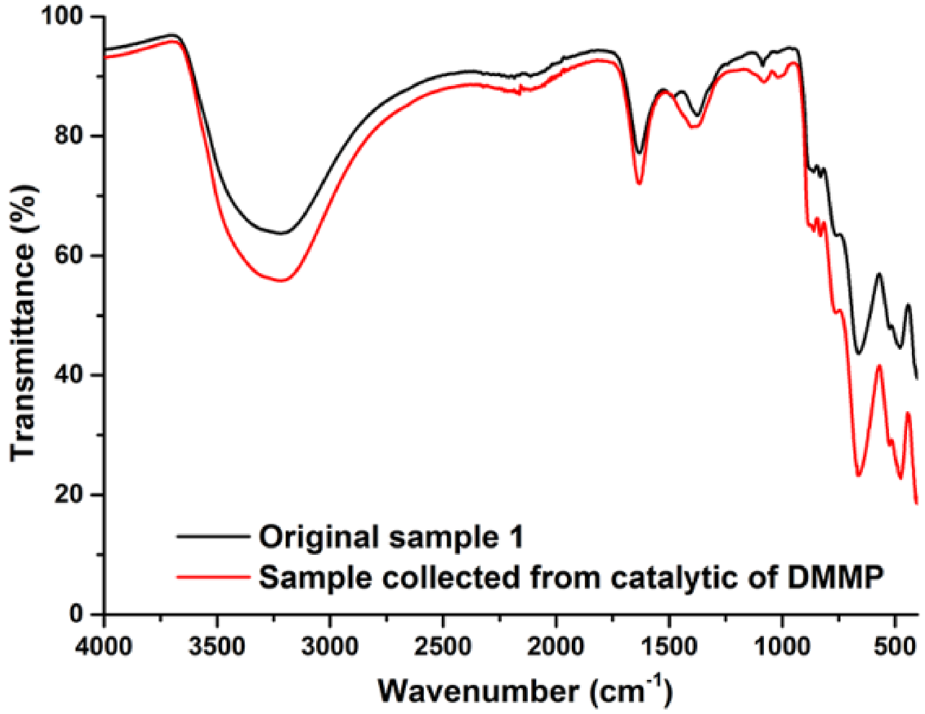

Fig. S34 IR spectra of **1** before and after catalytic hydrolysis of DMMP.

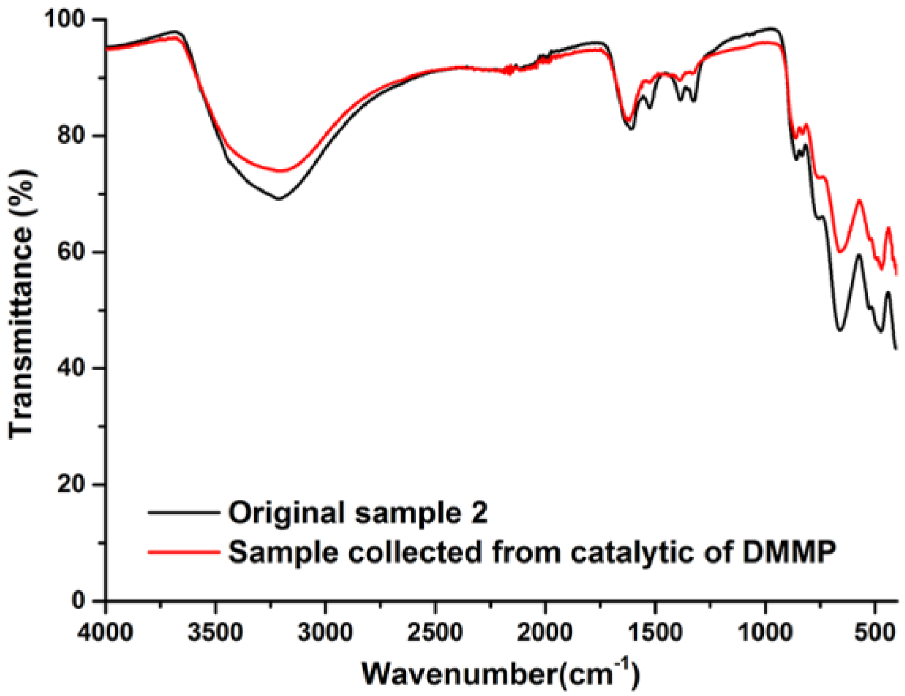

Fig. S35 IR spectra of **2** before and after catalytic hydrolysis of DMMP.

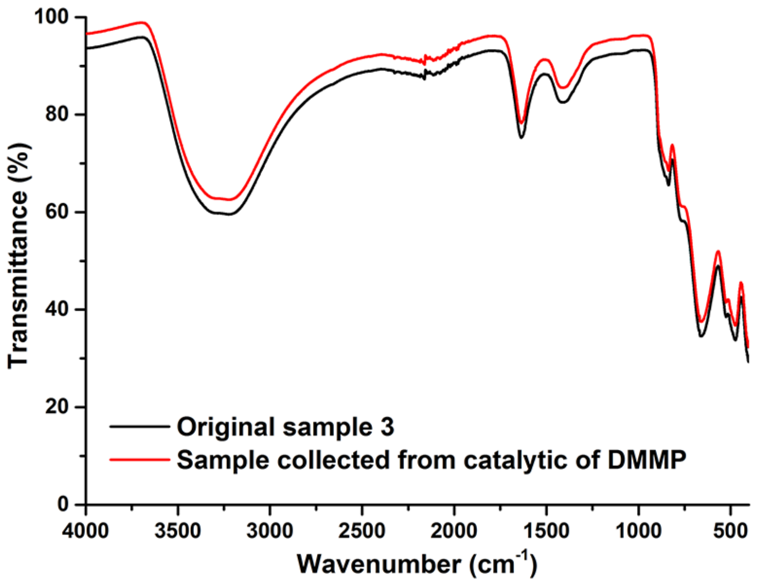

Fig. S36 IR spectra of **3** before and after catalytic hydrolysis of DMMP.

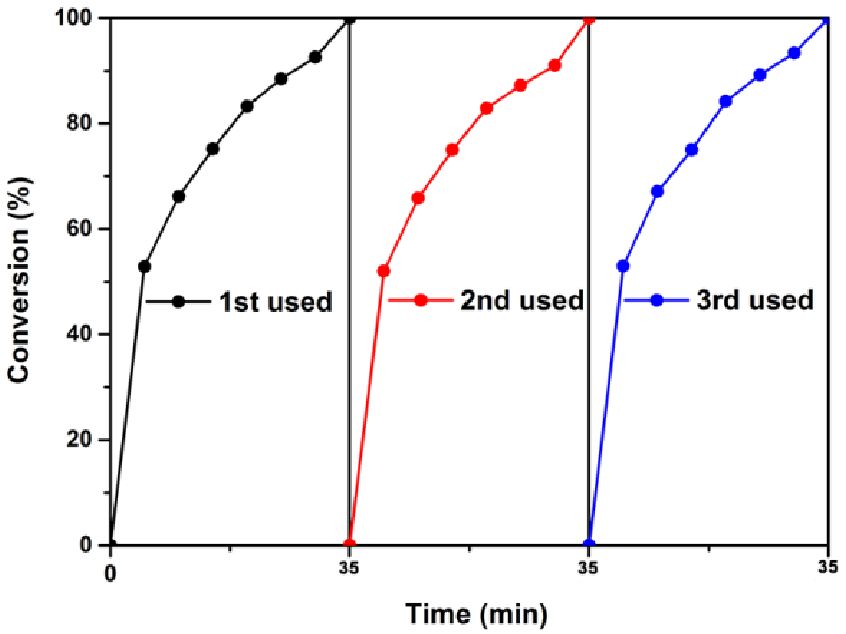

Fig. S37 Three-cycle conversions of DECP to DEHP versus reaction time of using catalysts **1**.

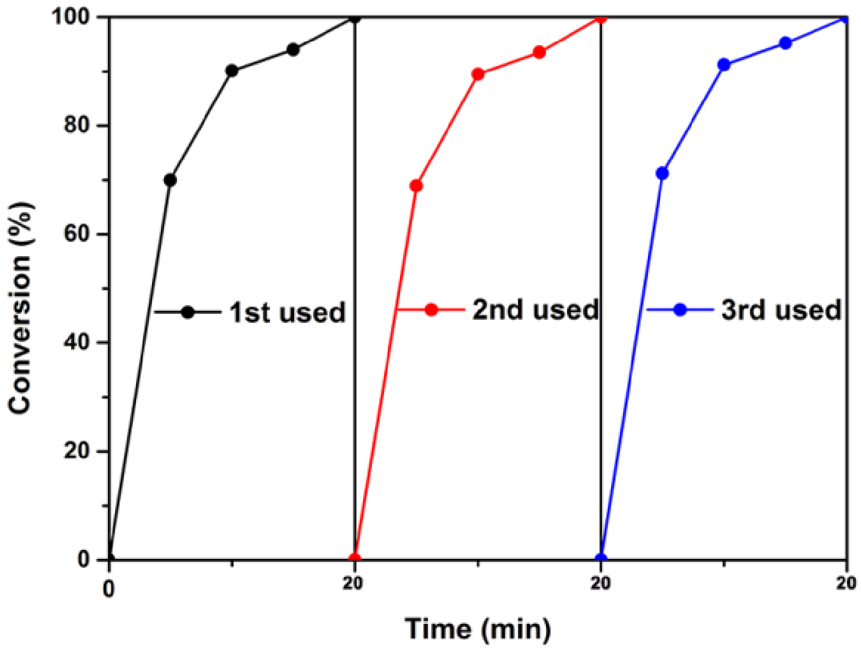

Fig. S38 Three-cycle conversions of DECP to DEHP versus reaction time of using catalysts **2**.

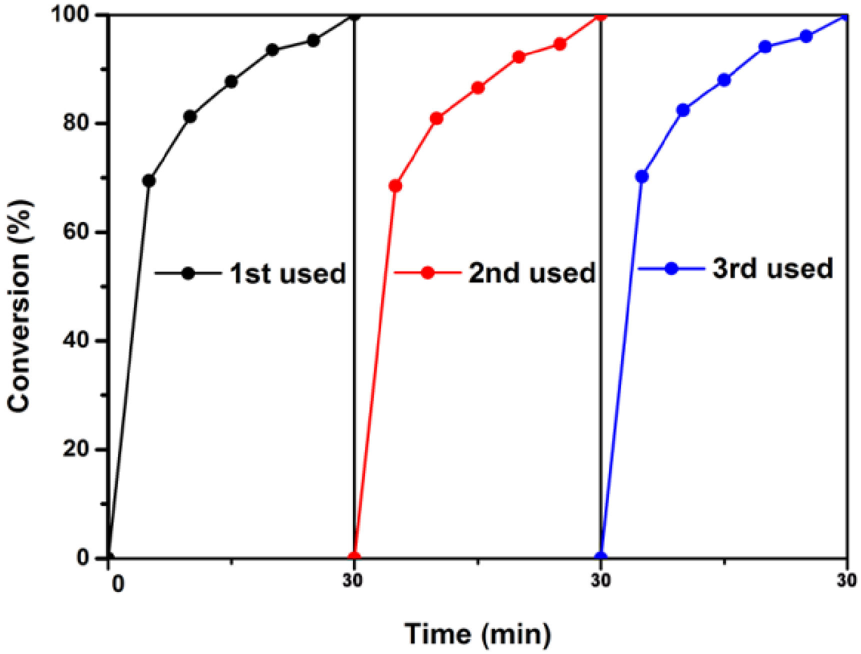

Fig. S39 Three-cycle conversions of DECP to DEHP versus reaction time of using catalysts **3**.

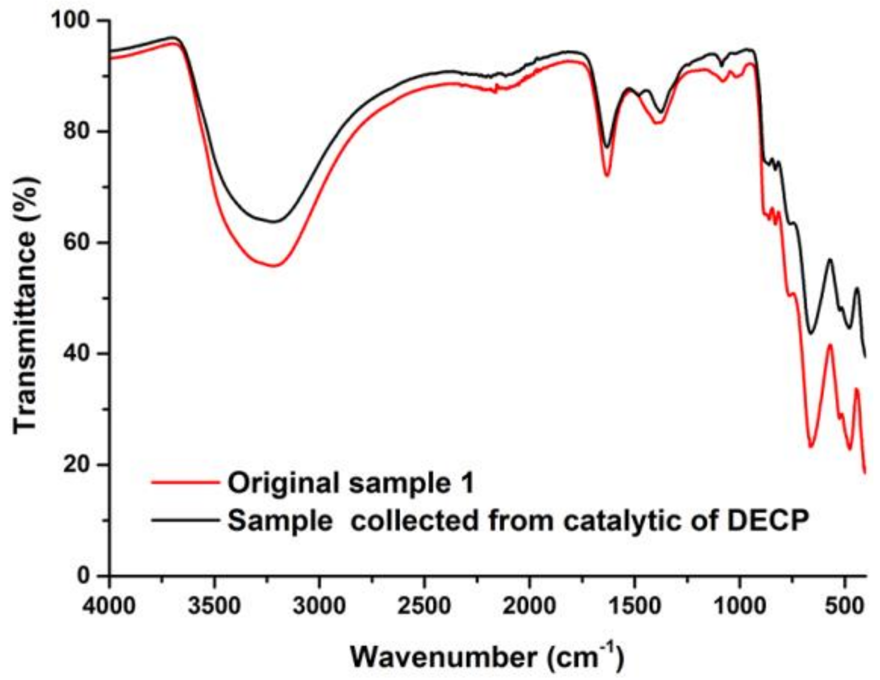

Fig. S40 IR spectra of **1** before and after catalytic hydrolysis of DECP.

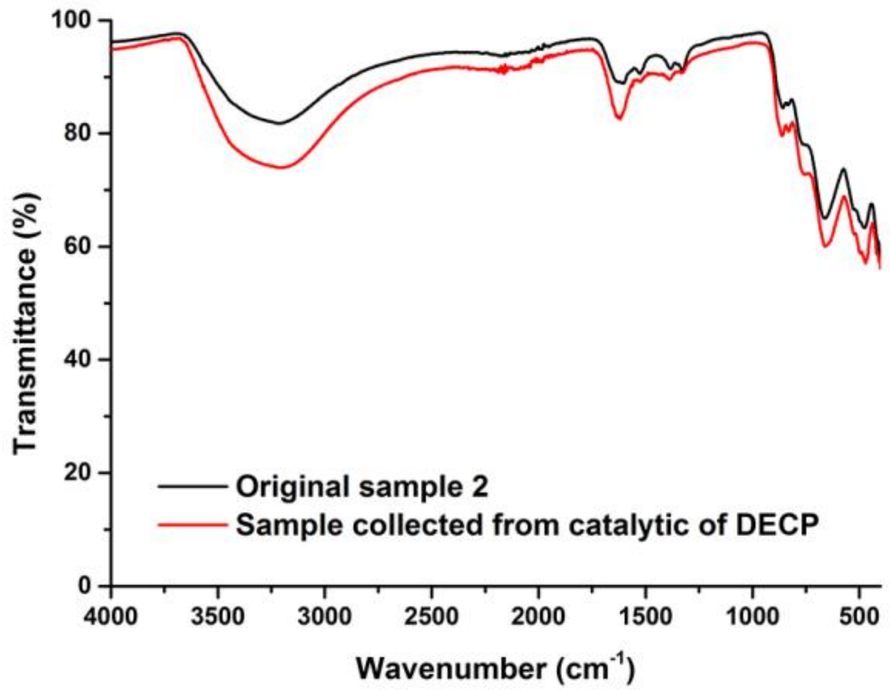

Fig. S41 IR spectra of **2** before and after catalytic hydrolysis of DECP.

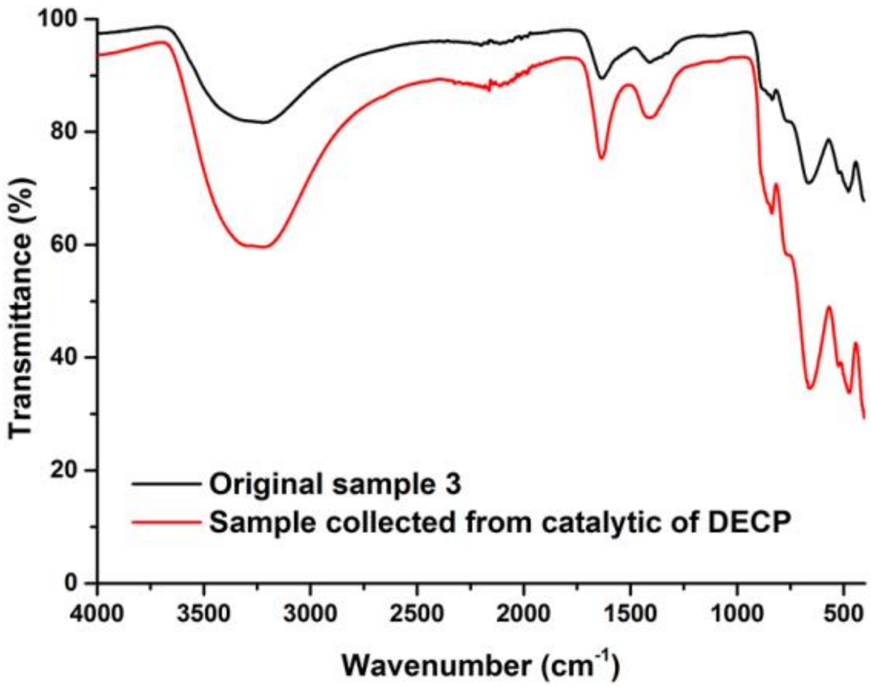

Fig. S42 IR spectra of **3** before and after catalytic hydrolysis of DECP.

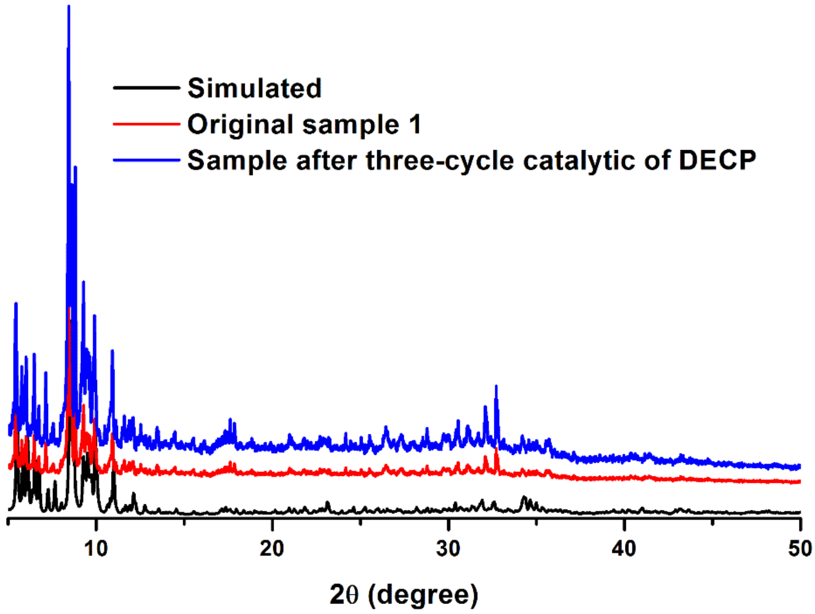

Fig. S43 XRD patterns of **1** after three-cycle catalytic hydrolysis of DECP.

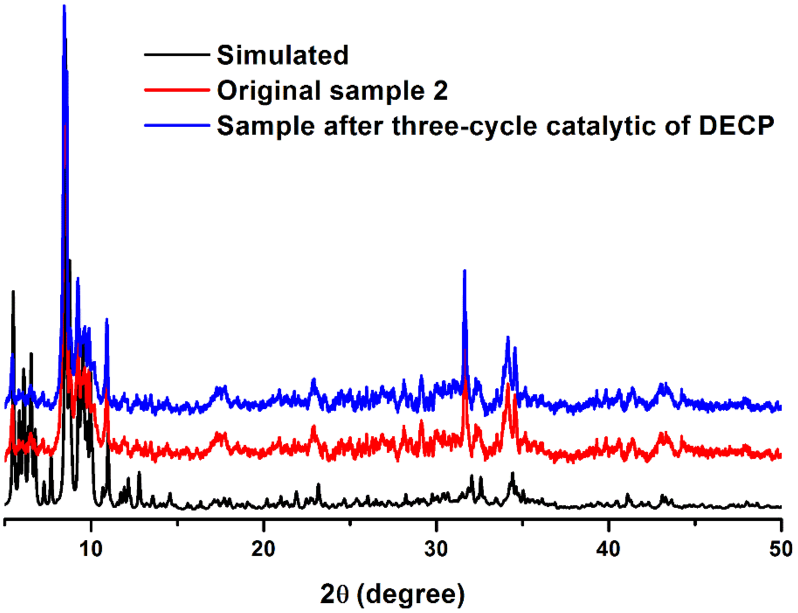

Fig. S44 XRD patterns of **2** after three-cycle catalytic hydrolysis of DECP.

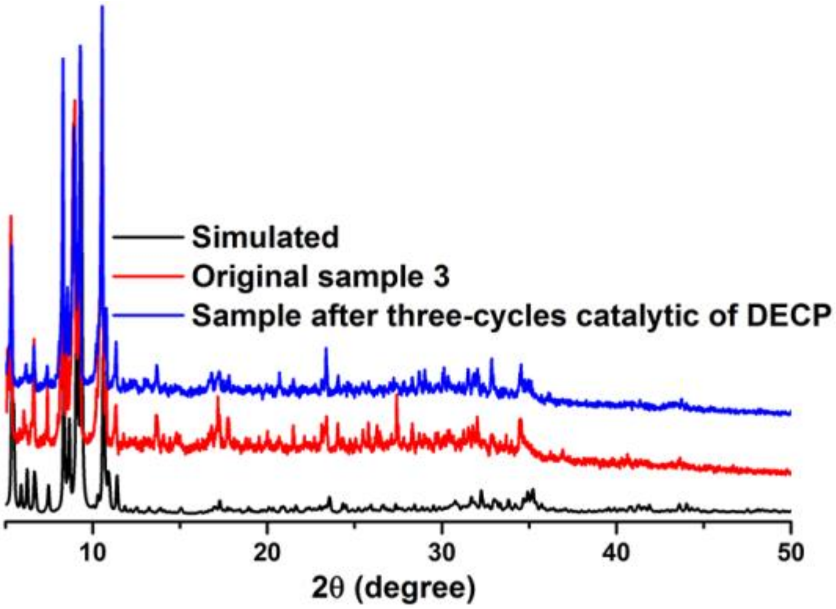

Fig. S45 XRD patterns of **3** after three-cycle catalytic hydrolysis of DECP.

References

[S1]. FILOWITZ, M., HO, R. K. C., KLEMPERER, W. G., and SHUM, W. (1979). ^{17}O Nuclear Magnetic Resonance Spectroscopy of Polyoxometalates. 1. Sensitivity and Resolution. *Inorg. Chem.* 18, 93-103.

[S2]. Shen, J. Q., Zhang Y., Zhang, Z. M., Li, Y.G., Gao, Y. Q. and Wang, E. B. (2014). Polyoxoniobate-based 3D framework materials with photocatalytic hydrogen evolution activity. *Chem. Commun*. 50, 6017-6019. doi:

org/10.1039/C3CC49245A
[S3]. Nyman, M., Bonhomme, F., Alam, T. M., Rodriguez, M. A., Cherry, B. R., Krumhansl, J. L., Nenoff, T. M. and Sattler, A. M. (2002). A general synthetic procedure for heteropolyniobates. *Science*. 297, 996-998. doi: 10.1126/science.1073979
[S4]. Guo, G., Xu, Y., Cao, J. and Hu, C. (2012). The {$V_4Nb_6O_{30}$} Cluster: A new type of vanadoniobate anion structure. *Chem. Eur. J.* 18, 3493-3497. doi: org/10.1002/chem.201103390
[S5]. Son, J. H., Ohlin, C. A., Johnson, R. L., Yu, P. and Casey, W. H. (2013). A soluble phosphorus-centered Keggin polyoxoniobate with bicapping vanadyl groups. *Chem. Eur. J.* 19, 5191-5197. doi: org/10.1002/chem.201204563
[S6]. Chris, R., Valérie, B., Evan, G. M., Christian, R., Colette, B. (2012). Sensitization of lanthanoid luminescence by organic and inorganic ligands in lanthanoid-organic-polyoxometalates. *Inorg. Chem.* 51, 1142-1151. doi: org/10.1021/ic202349u
[S7]. Sheldrick, G. M. (1997). *SHELXL97,* Program for Crystal Structure Refinement, University of Göttingen: Göttingen, Germany.
